# Tobacco Product Use and Associated Factors Among Middle and High School Students — National Youth Tobacco Survey, United States, 2021

**DOI:** 10.15585/mmwr.ss7105a1

**Published:** 2022-03-11

**Authors:** Andrea S. Gentzke, Teresa W. Wang, Monica Cornelius, Eunice Park-Lee, Chunfeng Ren, Michael D. Sawdey, Karen A. Cullen, Caitlin Loretan, Ahmed Jamal, David M. Homa

**Affiliations:** ^1^Office on Smoking and Health, National Center for Chronic Disease Prevention and Health Promotion, CDC; ^2^Center for Tobacco Products, Food and Drug Administration

## Abstract

**Problem/Condition:**

Commercial tobacco use is the leading cause of preventable disease, disability, and death in the United States. Most tobacco product use begins during adolescence. In recent years, tobacco products have evolved to include various combusted, smokeless, and electronic products.

**Period Covered:**

2021.

**Description of System:**

The National Youth Tobacco Survey (NYTS) is an annual, cross-sectional, school-based, self-administered survey of U.S. middle school (grades 6–8) and high school (grades 9–12) students. A three-stage cluster sampling procedure is used to generate a nationally representative sample of U.S. students attending public and private schools. NYTS is the only nationally representative survey of U.S. middle and high school students that focuses exclusively on tobacco use patterns and associated factors. NYTS provides data to support the design, implementation, and evaluation of comprehensive youth tobacco use prevention and control programs and to guide tobacco regulatory activities. Since 2019, NYTS has been administered electronically via tablet computers. Because of emergency COVID-19 protocols that were in place across the United States during the 2021 NYTS fielding window (January 18–May 21, 2021), the 2021 survey was administered using a web URL to allow participation by eligible students learning under varying instructional models (in-person, distance/virtual, and hybrid). In total, 50.8% of student respondents reported completing the survey in a school building or classroom and 49.2% at home or some other place. CDC and the Food and Drug Administration (FDA) analyzed data from the 2021 NYTS to assess tobacco product use patterns and associated factors among U.S. middle and high school students. Overall, 20,413 students (out of 25,149 sampled students; student response rate: 81.2%) completed the questionnaire from 279 schools (out of 508 sampled schools; school response rate: 54.9%). The overall response rate, defined as the product of the student and school response rates, was 44.6%. The sample was weighted to represent approximately 11.97 million middle school students and 15.44 million high school students. Students with missing information about grade level were excluded from the school-level analyses (n = 135).

**Results:**

In 2021, an estimated 34.0% of high school students (5.22 million) and 11.3% of middle school students (1.34 million) reported ever using a tobacco product (i.e., electronic cigarettes [e-cigarettes], cigarettes, cigars, smokeless tobacco, hookahs, pipe tobacco, heated tobacco products, nicotine pouches, and bidis [small brown cigarettes wrapped in a leaf]). Current (past 30-day) use of a tobacco product was 13.4% for high school students (2.06 million) and 4.0% for middle school students (470,000). E-cigarettes were the most commonly currently used tobacco product, cited by 11.3% of high school students (1.72 million) and 2.8% of middle school students (320,000), followed by cigarettes, cigars, smokeless tobacco, hookahs, nicotine pouches, heated tobacco products, and pipe tobacco. Current use of any tobacco product was reported by 14.2% of students identifying as lesbian, gay, or bisexual (LGB) (versus 7.9% of heterosexual); 18.9% of students identifying as transgender (versus 8.2% of not transgender); and 14.2% of students reporting severe psychological distress (versus 5.5% with no distress). Among students who currently used each respective tobacco product, frequent use (on ≥20 days of the past 30 days) ranged from 17.2% for nicotine pouches to 39.4% for e-cigarettes. Among current users of any tobacco product, 79.1% reported using a flavored tobacco product; by product, e-cigarettes were the most commonly used flavored tobacco product. Among current users of any tobacco product, the most commonly reported source of access was from a friend (32.8%). Among students who currently used e-cigarettes, 53.7% used a disposable device, 28.7% used a prefilled/refillable pod or cartridge device, 9.0% used a tank or mod system (a system that can be customized by the user), and 8.6% did not know the device type. Among students who had ever used e-cigarettes, the most common reason for first trying them was “a friend used them” (57.8%); among current e-cigarette users, the most commonly cited reason for current use was “I am feeling anxious, stressed, or depressed” (43.4%). Among all middle and high school students, 75.2% reported past-year recognition of any antitobacco public education campaign ads. Exposure to marketing or advertising for any tobacco product was reported by 75.7% of students who had contact with an assessed potential source of tobacco product advertisements or promotions (going to a convenience store, supermarket, or gas station; using the Internet; watching television or streaming services or going to the movies; or reading newspapers or magazines). Among students who reported using social media, 73.5% had ever seen e-cigarette–related content. Among all students, perceiving “no” or “little” harm from intermittent tobacco product use was highest for e-cigarettes (16.6%) and lowest for cigarettes (9.6%). Among students who currently used any tobacco product, 27.2% had experienced cravings during the past 30 days; 19.5% reported wanting to use a tobacco product within 30 minutes of waking. Moreover, 65.3% of students who currently used tobacco products were seriously thinking about quitting the use of all products, and 60.2% had stopped using all products for ≥1 day because they were trying to quit during the past 12 months.

**Interpretation:**

In 2021, approximately one in 10 U.S. middle and high school students (9.3%) had used a tobacco product during the preceding 30 days. By school level, this represented more than one in eight high school students (13.4%) and approximately one in 25 middle school students (4.0%). E-cigarettes were the most commonly used tobacco product in 2021. Tobacco product use was higher among certain subpopulations, such as those identifying as LGB or transgender, or those reporting psychological distress. Importantly, approximately two thirds of students who currently used tobacco products were seriously thinking about quitting. However, factors that might continue to promote tobacco product use among U.S. youths, such as the availability of flavors, access to tobacco products, exposure to tobacco product marketing, and misperceptions about harm from tobacco product use, remained prevalent in 2021.

**Public Health Action:**

The continued monitoring of all forms of youth tobacco product use and associated factors through surveillance efforts including NYTS is important to the development of public health policy and action at national, state, and local levels. The 2021 NYTS was successfully administered during the COVID-19 pandemic using a web URL to allow participation by eligible students learning under varying instructional models. As a result of these modifications to the fielding procedures, any comparison of results between 2021 NYTS findings with previous years, including the direct attribution of any potential changes in tobacco product use, is not possible. Parents, educators, youth advocates, and health care providers can help protect youths from the harms of tobacco products, including e-cigarettes. In addition, the comprehensive and sustained implementation of evidence-based tobacco control strategies, combined with FDA’s regulation of tobacco products, is important for reducing all forms of tobacco product use among U.S. youths.

## Introduction

Tobacco* product use is the leading cause of preventable disease, disability, and death in the United States (*1*). Preventing tobacco product use among youths is critical to decreasing morbidity and mortality because almost all tobacco product use begins during adolescence or young adulthood; approximately nine in 10 adults who smoke cigarettes started before age 18 years (*1*–*3*). In recent years, tobacco products have evolved to include various combusted, smokeless, and electronic products.

The National Youth Tobacco Survey (NYTS), conducted periodically during 1999–2009 and annually since 2011, provides national data on estimates of tobacco product use to support the design, implementation, and evaluation of comprehensive youth tobacco prevention and control programs and to inform tobacco regulatory activities in the United States (*4*). NYTS is the only nationally representative survey of U.S. middle (grades 6–8) and high school (grades 9–12) students that focuses exclusively on tobacco product use.

This report presents findings from the 2021 NYTS and describes the prevalence of youth tobacco product use and associated factors, including flavored tobacco product use, reasons for use, access to tobacco products, exposure to pro-tobacco and antitobacco product marketing, harm perceptions, urges to use tobacco products, and quitting behaviors. These findings can be used by public health professionals, health care providers, policymakers, parents, and other youth advocates to prevent and reduce tobacco product use among U.S. youths.

## Methods

### NYTS Sampling Procedures

NYTS is a cross-sectional, school-based, self-administered survey of U.S. middle and high school students (*4*). The 2021 NYTS sampling frame consisted of all regular public and private schools with students enrolled in grades 6–12 in the 50 U.S. states and the District of Columbia. The sampling frame comprised data obtained from Market Data Retrieval (*5*) and the National Center for Education Statistics (*6*,*7*). Alternative schools, special education schools, U.S. Department of Defense–operated schools, Bureau of Indian Affairs schools, vocational schools, and schools with a combined total of <40 students in grades 6–12 were excluded. Across 16 previous cycles of NYTS, school participation had averaged 80.8% with a low of 49.9%. Student participation had averaged 89.5% with a low of 85.9%. Historical participation rates at both school and student levels guided the sampling design and projected sample sizes. However, a more robust approach was used to calculate the projected sample sizes for the 2021 NYTS by assuming a more conservative overall response rate of 42.5%. The overall number of schools included in the sample was increased to account for higher levels of anticipated school refusals attributable to COVID-19 precautions. NYTS uses a stratified, three-stage cluster sample design (*4*). All students in the selected classes were eligible to participate in the survey; students who were unable to complete the questionnaire without special assistance were excluded.

### Data Collection and Processing

Because of emergency COVID-19 protocols across the United States, the 2021 NYTS was conducted using an online survey to allow eligible students to participate while at school or at home during a designated class period as part of a class activity.^†^ Participation in NYTS was voluntary at both the school and student levels; parental consent and student assent were required for NYTS participation. Students logged into a secure website from a school-issued or personal Internet-connected device, watched an instructional video, and responded to a question regarding their current location (e.g., classroom, home, or other location) before completing the survey. Data were transmitted directly to a secure server. Data collection procedures were supported by trained, off-site technical assistance providers who maintained regular contact with selected schools and teachers before, during, and after data collection activities. Students or whole classes that were unable to participate during the primary data collection period were asked to take the survey at the next possible opportunity. The 2021 NYTS was reviewed and approved by the Office of Management and Budget, the contracted data collectors’ institutional review board (IRB), and CDC’s IRB.^§^

The 2021 survey included 166 questions covering demographic information, tobacco product use behaviors, knowledge of and attitudes toward tobacco use, exposure to protobacco and antitobacco media and advertising, access to tobacco products, nicotine dependence, cessation attempts, exposure to secondhand smoke and e-cigarette aerosol, harm perceptions, exposure to tobacco product health warnings, and other tobacco-related topics. At the beginning of each tobacco product section, a description of the product, including example brands and generic images, was provided. Respondents did not answer all questions because of questionnaire skip patterns. Respondents could skip any question or end the survey at any time.

Survey administration occurred during January 18, 2021–May 21, 2021. The final sample consisted of 508 schools, of which 279 participated (school response rate: 54.9%); 20,413 student questionnaires were completed out of a sample of 25,149 students (student response rate: 81.2%). Student response rates varied by school instructional model; student response rates were 85.8% among schools with in-person instruction (n = 69 participating schools), 77.9% in schools with exclusive distance learning or virtual instruction (n = 68 participating schools), and 81.4% for schools with hybrid instruction (n = 142 participating schools). Hybrid student instructional models define instances in which some educational instruction happened in person and some happened virtually. This included various scenarios, including all students alternating between in-person and virtual learning or a portion of students receiving in-person instruction at all times and the remaining students receiving virtual instruction at all times. The overall response rate for the 2021 NYTS, defined as the product of the school-level and student-level response rates, was 44.6%. After exclusion of outliers, the average survey completion time was approximately 22 minutes. A weighting factor was applied to each student record to adjust for nonresponse and for varying probabilities of selection. Weights were adjusted to ensure that the weighted proportions of students in each grade matched national population proportions. Additional information on the NYTS sampling design, recruitment procedures, and data weighting is available (https://www.cdc.gov/tobacco/data_statistics/surveys/nyts/index.htm).

### Measures

#### Ever and Current Tobacco Product Use

Nine tobacco products were assessed: electronic cigarettes (e-cigarettes), cigarettes, cigars (cigars, cigarillos, and little cigars), smokeless tobacco (chewing tobacco, snuff, dip, snus, and dissolvable tobacco products), hookahs, pipe tobacco, bidis (small brown cigarettes wrapped in a leaf), heated tobacco products (HTPs), and nicotine pouches (pouches containing nicotine powder that comes from tobacco, which users place in their mouth). For each product, ever use was defined as ever using the product, and current use was defined as use on ≥1 day during the past 30 days. Any tobacco product use was defined as use of one or more of the nine tobacco products. Use of two or more tobacco products was defined as use of two or more of the nine tobacco products. Any combustible tobacco product use was defined as use of one or more of the following: cigarettes, cigars, hookahs, pipe tobacco, and bidis.

#### Demographic Factors

Demographic covariates assessed included sex (female or male), race and ethnicity (non-Hispanic White, non-Hispanic Black, Hispanic, or non-Hispanic other race), sexual identity (heterosexual; lesbian, gay, or bisexual [LGB]; or not sure), and whether the respondent identified as transgender (no, yes, not sure, or don’t know what this question is asking).

#### Social Determinant Indicators

Social determinant indicators included academic status in school (mostly As, Bs, Cs, Ds, or Fs), and speaking a language other than English at home (yes or no). Additionally, a composite scale made up of four questions was used to assess psychological distress: “During the past two weeks, how often have you been bothered by any of the following problems?” 1) “little interest or pleasure in doing things”; 2) “feeling down, depressed, or hopeless”; 3) “feeling nervous, anxious, or on edge”; and 4) “not being able or stop or control worrying.” For each item, response options were numerically coded (not at all = 0; several days = 1; more than half of the days = 2; nearly every day = 3), summed (range: 0–12), and categorized as none (0–2), mild (3–5), moderate (6–8), or severe (9–12). Furthermore, a composite scale made up of four questions was used to assess family affluence. Numeric values were assigned to question responses as follows: 1) “Does your family own a vehicle (such as a car, van, or truck)?” [no = 0; yes, one = 1; yes, two or more = 2]; 2) “Do you have your own bedroom?” [no = 0; yes = 1]; 3) “How many computers (including laptops and tablets, not including game consoles and smartphones) does your family own?” [none = 0; one = 1; two = 2; more than two = 3]; and 4) “During the past 12 months, how many times did you travel on vacation with your family?” [not at all = 0; once = 1; twice = 2; more than twice = 3]. Responses were summed (range: 0–9) and categorized into approximate tertiles based on the weighted distribution of scores in this sample as low (0–5), medium (6–7), and high (8–9).

#### Frequency of Tobacco Product Use

Respondents who reported ever use of any tobacco product were asked, “During the past 30 days, on how many days did you [use e-cigarettes; smoke cigarettes; smoke cigars, cigarillos, or little cigars; use chewing tobacco, snuff, or dip; use snus; use dissolvable tobacco products; smoke tobacco in a hookah or water pipe; smoke pipes filled with tobacco; use an HTP; or use a nicotine pouch].” Response options ranged between 0 and 30 days. Response options were categorized as 1–5 days, 6–19 days, and 20–30 days. Frequent use was defined as using a product on ≥20 days of the past 30 days.

#### Flavored Tobacco Product Use

For each tobacco product (excluding cigarettes), current users were asked, “Were any of the [tobacco product] that you used in the past 30 days flavored to taste like menthol, mint, clove or spice, alcoholic drinks, candy, fruit, chocolate, or any other flavor?” Response options were “yes,” “no,” and “don’t know.” Those who responded “yes” were categorized as a current flavored product user. Current flavored tobacco product users were asked, “What flavors were the [tobacco product] that you have used in the past 30 days? (Select one or more).” Response options were “menthol,” “mint,” “clove or spice,” “fruit,” “chocolate,” “alcoholic drinks (such as wine, margarita, or other cocktails),” “candy, desserts, or other sweets,” and “some other flavor not listed here.” Those who selected “some other flavor not listed here” could provide a write-in response; write-in responses corresponding to an original response option were recoded.

For cigarettes, current users were categorized as flavored (menthol) cigarette smokers if they responded “yes” to the question, “Menthol cigarettes are cigarettes that taste like mint. During the past 30 days, were the cigarettes that you usually smoked menthol?” or if they indicated “Kool” or “Newport” as their usual cigarette brand during the past 30 days. Usual cigarette brand was determined based on responses to two questions: “During the past 30 days, what brands of cigarettes did you smoke? (Select one or more)” and “During the past 30 days, what brand of cigarettes did you usually smoke? (Choose only one answer).” If “Kool” or “Newport” was the only brand selected for the first question, or if multiple brands were selected in the first question and “Kool” or “Newport” was selected for the second question, “Kool” or “Newport” was considered the respondent’s usual brand.

#### E-Cigarette Device Type

Respondents who reported current use of e-cigarettes were asked, “Which of the following best describes the type of e-cigarette you have used in the past 30 days? If you have used more than one type, please think about the one you use most often.” Response options were “A disposable e-cigarette (for example, Puff Bar or Stig),” “An e-cigarette that uses pre-filled or refillable pods or cartridges (for example, Juul, Smok, or Suorin),” “An e-cigarette with a tank that you refill with liquids (including mod systems that can be customized by the user),” and “I don’t know the type.”

#### Reasons for E-Cigarette Use

Respondents who had ever used e-cigarettes were asked, “Why did you first use an e-cigarette?” Respondents who reported current e-cigarette use were further asked, “Why do you currently use e-cigarettes?” For both questions, respondents could select one or more of 14 specified reasons. Respondents who indicated, “I used/use them for some other reasons” to either question could specify a write-in response; analyses of write-in responses were not included in this report.

#### Access to Tobacco Products

For each tobacco product, access sources were assessed by two questions: “During the past 30 days, how did you get your [tobacco product]?” (respondents could select one or more of eight specified responses) and “During the past 30 days, where did you buy your [tobacco product]?” (respondents could select one or more of 12 specified responses). Respondents could specify a write-in response; analyses of write-in responses were not included in this report.

#### Recognition of Public Education Campaigns Against Tobacco Product Use

All respondents were asked two questions regarding public education campaigns against tobacco product use: “In the past 12 months, have you seen or heard The Real Cost, on television, the Internet, social media, or radio as part of ads about tobacco?” (response options were yes, no, or not sure) and “In the past 12 months, have you seen or heard any other ads against tobacco with the following names or slogans on television, the Internet, social media, or on the radio?” Respondents could select one or more of “Truth,” “Tips or Tips from Former Smokers,” “Fresh Empire,” “This Free Life,” or “some other ad” or could select “I haven’t seen or heard any of these ads.” Those who indicated having seen “some other ad” could specify with a write-in response; analyses of write-in responses were not included in this report. A composite of having recognized at least one ad against tobacco product use from a specified response option also was generated.

#### Exposure to Tobacco Product Marketing

Exposure to tobacco product marketing (advertisements or promotions) was assessed separately for e-cigarettes and cigarettes or other tobacco products for four sources: retail stores; Internet; television, streaming services, or movies; and newspapers or magazines. Respondents were asked, “When you [are using the Internet; read newspapers or magazines; go to a convenience store, supermarket, or gas station; watch television or streaming services (such as Netflix, Hulu, or Amazon Prime), or go to the movies], how often do you see ads or promotions for [e-cigarettes; cigarettes or other tobacco products]?” Respondents were categorized as exposed if they responded “sometimes,” “most of the time,” or “always” or unexposed if they responded “never” or “rarely.” Those who reported “I do not use the Internet,” “I do not read newspapers or magazines,” “I never go to a convenience stores, supermarket, or gas station,” or “I do not watch TV or streaming services or go to the movies” were excluded from the analysis.

#### E-Cigarette Content on Social Media

All respondents were asked, “How often do you use social media?” Respondents who reported a response other than “never/I don’t use social media” were asked, “When you use social media, how often 1) “do you see posts or content (pictures, videos, or text) related to e-cigarettes?”; 2) “do you post pictures or videos of yourself or someone else using e-cigarettes?”; and 3) “have you liked, commented, or shared posts or content (pictures, videos, or text) related to e-cigarettes?” For each question, response options were “never,” “less than monthly,” “monthly,” “weekly,” or “daily.”

#### Harm Perceptions

The 2021 NYTS assessed harm perceptions for five tobacco products: e-cigarettes; cigarettes; cigars, cigarillos, or little cigars; smokeless tobacco (chewing tobacco, snuff, dip, snus, or dissolvable tobacco products); and hookahs. All respondents were asked, “How much do you think people harm themselves when they (use e-cigarettes; smoke cigarettes; smoke cigars, cigarillos, or little cigars; use chewing tobacco, snuff, dip, snus, or dissolvable tobacco products; or smoke tobacco in a hookah or water pipe) some days but not every day?” Response options were “no harm,” “little harm,” “some harm,” and “a lot of harm.”

#### Dependence and Cessation Indicators

##### Urges to Use Tobacco Products

Students who currently used any tobacco product were asked, “During the past 30 days, have you had a strong craving or felt like you really needed to use a tobacco product of any kind?” (yes or no); and “How soon after you wake up do you want to use a tobacco product of any kind?” Response options were dichotomized as wanting to use a tobacco product within 30 minutes (“within 5 minutes” or “from 6 to 30 minutes,”) or not (“from more than 30 minutes to 1 hour,” “after more than 1 hour but less than 24 hours,” “I rarely want to use tobacco products,” or “I do not want to use tobacco products”).

##### Quitting Behaviors

Students who currently used any tobacco product were asked, “Are you seriously thinking about quitting the use of all tobacco products?” Responses were dichotomized as yes (“yes, during the next [30 days; 6 months; 12 months]” or “yes, but not during the next 12 months”) or no (“no, I am not thinking about quitting the use of all tobacco products”). Students who currently used any tobacco product were asked, “During the past 12 months, how many times have you stopped using all tobacco products for one day or longer because you were trying to quit all tobacco products for good?” Responses were dichotomized as making a past-year quit attempt (“1 time,” “2 times,” “3–5 times,” “6–9 times,” or “10 or more times”) or not making an attempt (“I did not try to quit all tobacco products during the past 12 months”).

### Analyses

Statistical analyses were conducted by using SAS-callable SUDAAN software (version 11.0.1; RTI International) to account for the complex sampling design. Weighted prevalence estimates and 95% CIs were computed for all measures; when applicable, population totals were estimated from probability weights. Results with unweighted denominators <50 or a relative standard error >30% are not reported. Comparisons of estimates from the 2021 NYTS with previous years were not possible because of the modifications to the fielding procedures in 2021 in response to the COVID-19 pandemic. The 20,413 student records were weighted to represent approximately 27.56 million students. On the basis of self-reported grade level, this included 9,763 middle school students (11.97 million) and 10,515 high school students (15.44 million); 135 students with missing information on grade level were excluded from school-level analyses. Overall, 50.8% of student respondents reported completing the survey in a school building or classroom and 49.2% at home or some other place.

## Results

### Ever Tobacco Product Use

In 2021, among U.S. middle and high school students, 24.1% (6.6 million) reported ever use of a tobacco product (high school: 34.0%, 5.22 million; middle school: 11.3%, 1.34 million) (Table 1). Among students who ever used a tobacco product, 49.4% (3.2 million) had ever used a combustible tobacco product and 41.5% (2.74 million) had ever used two or more tobacco product types. E-cigarettes were the most commonly ever used tobacco product overall (19.4%; 5.3 million), followed by cigarettes (8.1%), cigars (5.2%), smokeless tobacco (3.6%), hookahs (2.8%), nicotine pouches (1.9%), HTPs (1.8%), and pipe tobacco (1.0%). Ever use of any tobacco product was reported by 28.0% of middle and high school students who reported taking the survey in a school or classroom and 20.5% of middle and high school students who reported taking the survey at home or some other place (data not shown). 

**TABLE 1:** Percentage of middle and high school students who reported ever using tobacco products, by product,* overall and by school level, sex, and race and ethnicity — National Youth Tobacco Survey, United States, 2021

Characteristic	Sex		Race and ethnicity				Total	
	Female	Male	White, non-Hispanic	Black, non-Hispanic	Hispanic^†^	Other, non-Hispanic		
	% (95% CI)	% (95% CI)	% (95% CI)	% (95% CI)	% (95% CI)	% (95% CI)	% (95% CI)	Estimated no. of users^§^
**Overall**								
**Any tobacco product^¶^**	**24.5 (22.2–27.1)**	**23.8 (21.7–26.1)**	**26.7 (23.7–29.9)**	**21.9 (19.2–24.9)**	**22.4 (20.7–24.2)**	**16.1 (11.6–21.8)**	**24.1 (22.1–26.3)**	**6,600,000**
E-cigarettes	20.1 (17.9–22.5)	18.8 (16.8–21.0)	23.1 (20.3–26.2)	12.6 (10.4–15.2)	17.6 (15.8–19.5)	12.7 (8.9–17.7)	**19.4 (17.5–21.5)**	**5,300,000**
Cigarettes	8.0 (6.9–9.1)	8.3 (7.3–9.4)	9.3 (8.1–10.8)	6.2 (4.8–8.0)	7.1 (6.0–8.4)	5.9 (3.5–10.0)	**8.1 (7.2–9.1)**	**2,180,000**
Cigars	3.7 (3.2–4.3)	6.6 (5.6–7.9)	5.9 (4.7–7.3)	6.8 (5.5–8.4)	4.1 (3.4–4.9)	1.9 (1.1–3.4)	**5.2 (4.5–6.0)**	**1,400,000**
Smokeless tobacco	2.2 (1.6–2.8)	5.0 (4.1–6.2)	4.8 (3.9–5.8)	1.8 (1.2–2.7)	2.3 (1.7–3.0)	—**	**3.6 (2.9–4.5)**	**960,000**
Hookahs	3.1 (2.5–3.8)	2.5 (2.0–3.0)	2.1 (1.6–2.7)	5.0 (4.0–6.3)	3.2 (2.3–4.3)	—	**2.8 (2.3–3.3)**	**740,000**
Nicotine pouches	1.2 (0.9–1.7)	2.6 (2.0–3.2)	2.6 (2.1–3.3)	0.7 (0.4–1.3)	1.3 (0.9–1.9)	—	**1.9 (1.5–2.4)**	**490,000**
Heated tobacco products	1.8 (1.5–2.2)	1.7 (1.5–2.0)	1.7 (1.4–2.1)	1.7 (1.1–2.5)	2.1 (1.5–2.9)	—	**1.8 (1.5–2.0)**	**440,000**
Pipe tobacco	0.9 (0.6–1.2)	1.2 (0.9–1.6)	1.3 (1.0–1.6)	—	0.9 (0.7–1.3)	—	**1.0 (0.8–1.3)**	**270,000**
Any combustible tobacco product^††^	11.3 (10.1–12.5)	12.4 (11.1–13.8)	12.6 (10.9–14.4)	14.0 (11.8–16.4)	10.4 (9.3–11.6)	8.3 (5.5–12.4)	**11.9 (10.8–13.0)**	**3,200,000**
Two or more tobacco products^§§^	9.1 (8.0–10.3)	10.9 (9.5–12.4)	11.8 (10.1–13.7)	8.5 (6.8–10.5)	8.5 (7.3–9.9)	6.3 (4.2–9.4)	**10.0 (8.9–11.3)**	**2,740,000**
**High school (grades 9–12)**								
**Any tobacco product**	**35.2 (32.4–38.1)**	**33.0 (30.3–35.8)**	**37.8 (34.7–41.1)**	**27.7 (25.1–30.6)**	**29.7 (27.2–32.4)**	**27.9 (22.5–34.0)**	**34.0 (31.6–36.5)**	**5,220,000**
E-cigarettes	30.2 (27.4–33.1)	27.7 (25.0–30.6)	33.8 (30.7–37.1)	16.9 (14.5–19.7)	25.0 (22.4–27.9)	23.0 (17.6–29.5)	**28.9 (26.4–31.4)**	**4,430,000**
Cigarettes	10.8 (9.3–12.5)	11.5 (9.9–13.3)	13.0 (11.3–14.9)	6.4 (4.7–8.5)	9.5 (7.6–11.9)	9.6 (5.6–15.8)	**11.2 (9.9–12.6)**	**1,680,000**
Cigars	4.9 (4.2–5.8)	10.0 (8.6–11.7)	8.7 (7.3–10.4)	8.7 (7.0–10.6)	5.4 (4.3–6.8)	—	**7.6 (6.6–8.8)**	**1,140,000**
Smokeless tobacco	2.4 (1.8–3.3)	6.8 (5.5–8.4)	6.6 (5.4–7.9)	1.7 (1.0–2.9)	2.2 (1.5–3.2)	—	**4.7 (3.8–5.9)**	**700,000**
Hookahs	4.2 (3.4–5.3)	3.5 (2.8–4.3)	2.9 (2.3–3.8)	6.8 (5.3–8.7)	4.0 (3.0–5.3)	5.1 (2.9–8.9)	**3.8 (3.2–4.6)**	**570,000**
Nicotine pouches	1.7 (1.3–2.4)	4.1 (3.3–5.1)	4.1 (3.3–5.1)	—	1.5 (1.0–2.2)	—	**3.0 (2.4–3.6)**	**420,000**
Heated tobacco products	2.5 (1.9–3.1)	2.1 (1.8–2.5)	2.3 (1.9–2.8)	2.1 (1.2–3.7)	2.4 (1.6–3.4)	—	**2.3 (2.0–2.6)**	**310,000**
Pipe tobacco	1.1 (0.7–1.8)	1.7 (1.2–2.4)	1.8 (1.3–2.4)	—	1.1 (0.7–1.8)	—	**1.4 (1.1–1.9)**	**210,000**
Any combustible tobacco product	15.4 (13.6–17.2)	17.6 (15.9–19.5)	17.8 (15.9–19.8)	17.4 (15.0–20.1)	13.5 (11.3–16.2)	14.1 (9.8–19.9)	**16.6 (15.1–18.1)**	**2,500,000**
Two or more tobacco products	12.8 (11.4–14.4)	16.2 (14.3–18.4)	17.2 (15.2–19.4)	10.8 (8.8–13.4)	11.6 (9.5–14.1)	10.9 (7.6–15.5)	**14.6 (13.1–16.3)**	**2,240,000**
**Middle school (grades 6–8)**								
**Any tobacco product**	**11.1 (9.3–13.2)**	**11.5 (9.9–13.4)**	**10.3 (8.7–12.1)**	**14.0 (10.9–17.8)**	**13.2 (10.5–16.5)**	**6.0 (3.7–9.6)**	**11.3 (9.8–13.0)**	**1,340,000**
E-cigarettes	7.5 (6.2–9.1)	7.0 (5.8–8.5)	7.5 (6.3–9.0)	7.0 (4.9–9.8)	8.4 (6.5–10.8)	3.8 (2.2–6.6)	**7.3 (6.2–8.6)**	**860,000**
Cigarettes	4.2 (3.2–5.6)	3.9 (3.1–5.0)	3.8 (2.9–5.1)	5.7 (3.8–8.4)	4.1 (2.8–5.8)	—	**4.1 (3.2–5.1)**	**480,000**
Cigars	2.0 (1.5–2.7)	2.2 (1.7–2.7)	1.7 (1.2–2.3)	4.3 (3.2–5.8)	2.5 (1.6–3.8)	—	**2.1 (1.7–2.6)**	**240,000**
Smokeless tobacco	1.8 (1.2–2.6)	2.5 (1.8–3.6)	2.1 (1.5–2.8)	1.9 (1.1–3.3)	2.3 (1.5–3.5)	—	**2.2 (1.6–3.0)**	**250,000**
Hookahs	1.5 (1.0–2.3)	1.1 (0.7–1.5)	0.6 (0.4–1.0)	2.7 (1.8–3.9)	2.1 (1.2–3.7)	—	**1.3 (0.9–1.8)**	**150,000**
Nicotine pouches	0.6 (0.3–1.1)	0.5 (0.3–0.8)	0.4 (0.3–0.7)	—	—	—	**0.6 (0.4–0.8)**	**60,000**
Heated tobacco products	1.0 (0.7–1.4)	1.2 (0.9–1.8)	0.8 (0.5–1.3)	—	1.7 (1.0–3.0)	—	**1.1 (0.9–1.5)**	**120,000**
Pipe tobacco	—	0.5 (0.3–0.8)	0.4 (0.2–0.6)	—	0.7 (0.4–1.2)	—	**0.5 (0.3–0.7)**	**50,000**
Any combustible tobacco product	6.0 (4.9–7.4)	5.4 (4.5–6.5)	4.7 (3.8–5.9)	9.2 (6.7–12.5)	6.5 (5.0–8.6)	—	**5.7 (4.8–6.8)**	**670,000**
Two or more tobacco products	4.3 (3.3–5.7)	3.7 (2.8–4.9)	3.6 (2.7–4.9)	5.4 (3.6–8.1)	4.7 (3.4–6.5)	—	**4.0 (3.2–5.1)**	**480,000**

**Abbreviation: **e-cigarettes = electronic cigarettes.

* Ever use of e-cigarettes was determined by asking, “Have you ever used an e-cigarette, even once or twice?” Ever use of cigarettes was determined by asking, “Have you ever smoked a cigarette, even one or two puffs?” Ever use of cigars was determined by asking, “Have you ever smoked a cigar, cigarillo, or little cigar, even one or two puffs?” Smokeless tobacco was defined as use of chewing tobacco, snuff, or dip, snus, or dissolvable tobacco products. Ever use of smokeless tobacco was determined by asking the following questions: for chewing tobacco, snuff, and dip: “Have you ever used chewing tobacco, snuff, or dip, even just a small amount?”; for snus: “Have you ever used snus, even just one time?”; and for dissolvable tobacco products: “Have you ever used dissolvable tobacco products, even just one time?” Responses from these questions were combined to derive overall smokeless tobacco use. Ever use of hookahs was determined by asking, “Have you ever smoked tobacco in a hookah or water pipe, even one or two puffs?” Ever use of nicotine pouches was determined by asking, “Have you ever used a “nicotine pouch,” even just one time?” Ever use of heated tobacco products was determined by asking, “Have you ever used a heated tobacco product, even just one time?” Ever use of pipe tobacco (not hookahs) was determined by asking, “Have you ever smoked pipes filled with tobacco (not hookah or water pipe), even just one time?” Because of missing data on the ever use questions, denominators for each tobacco product might be different. For each question, response options were “yes” or “no.”

† Hispanic persons could be of any race.

§ Estimated weighted total number of ever tobacco product users was rounded down to the nearest 10,000 persons. Overall estimates were reported among 20,413 U.S. middle and high school students. School level was determined by self-reported grade level: high school (grades 9–12; n = 10,515) and middle school (grades 6–8; n = 9,763). Overall estimates might not directly total to sums of corresponding subgroup estimates because of rounding or inclusion of students who did not self-report sex, race and ethnicity, or grade level.

¶ Any tobacco product use was defined as ever use of any tobacco product (e-cigarettes, cigarettes, cigars [cigars, cigarillos, or little cigars], smokeless tobacco [chewing tobacco, snuff, or dip, snus, or dissolvable tobacco products], hookahs, pipe tobacco, heated tobacco products, nicotine pouches, or bidis [small brown cigarettes wrapped in a leaf]), even just one time.

** Data were statistically unreliable because of unweighted denominator <50 or a relative standard error >30%.

†† Any combustible tobacco product use was defined as ever use of cigarettes, cigars (cigars, cigarillos, or little cigars), hookahs, pipe tobacco, or bidis, even just one time.

§§ Defined as ever use of two or more tobacco products (e-cigarettes, cigarettes, cigars [cigars, cigarillos, or little cigars], smokeless tobacco [chewing tobacco, snuff, or dip, snus, or dissolvable tobacco products], hookahs, pipe tobacco, heated tobacco products, nicotine pouches, or bidis), even just one time.

### Current Tobacco Product Use

Overall, among middle and high school students, 9.3% (2.55 million) reported current (past 30-day) use of any tobacco product (Table 2). Among students who currently used any tobacco product, 34.4% (860,000) currently used any combustible tobacco product and 29.0% (740,000) currently used two or more tobacco product types. E-cigarettes were the most commonly used tobacco product overall (7.6%; 2.06 million), followed by cigarettes (1.5%), cigars (1.4%), smokeless tobacco (0.9%), hookahs and nicotine pouches (both 0.8%), HTPs (0.7%), and pipe tobacco (0.3%). Current use of any tobacco product was reported by 11.7% of middle and high school students who reported taking the survey in a school or classroom and 6.9% of middle and high school students who reported taking the survey at home or some other place. 

**TABLE 2:** Percentage of middle and high school students who reported current (past 30-day) tobacco product use, by product,* overall and by school level, sex, and race and ethnicity — National Youth Tobacco Survey, United States, 2021

Characteristic	Sex		Race and ethnicity				Total	
	Female	Male	White, non-Hispanic	Black, non-Hispanic	Hispanic^†^	Other, non-Hispanic		
	% (95% CI)	% (95% CI)	% (95% CI)	% (95% CI)	% (95% CI)	% (95% CI)	% (95% CI)	Estimated no. of users^§^
**Overall**								
**Any tobacco product^¶^**	**9.6 (8.4–11.0)**	**9.0 (7.9–10.3)**	**11.0 (9.5–12.8)**	**8.2 (6.5–10.2)**	**7.4 (6.4–8.7)**	**5.4 (3.6–8.1)**	**9.3 (8.3–10.5)**	**2,550,000**
E-cigarettes	8.0 (6.8–9.4)	7.1 (6.1–8.3)	9.6 (8.2–11.4)	4.3 (3.1–6.0)	6.0 (4.9–7.2)	4.1 (2.7–6.2)	**7.6 (6.6–8.7)**	**2,060,000**
Cigarettes	1.5 (1.2–1.9)	1.5 (1.2–2.0)	1.8 (1.4–2.2)	1.0 (0.6–1.6)	1.5 (1.1–2.0)	—**	**1.5 (1.3–1.8)**	**410,000**
Cigars	1.1 (0.8–1.4)	1.8 (1.5–2.1)	1.4 (1.1–1.8)	3.1 (2.4–4.1)	0.9 (0.7–1.2)	—	**1.4 (1.2–1.7)**	**380,000**
Smokeless tobacco	0.5 (0.3–0.7)	1.3 (1.0–1.8)	1.1 (0.8–1.5)	—	0.7 (0.5–1.0)	—	**0.9 (0.7–1.2)**	**240,000**
Hookahs	0.9 (0.6–1.4)	0.8 (0.6–1.0)	0.5 (0.4–0.7)	2.2 (1.4–3.4)	1.0 (0.6–1.5)	—	**0.8 (0.7–1.1)**	**220,000**
Nicotine pouches	0.5 (0.3–0.7)	1.0 (0.7–1.4)	0.9 (0.6–1.3)	—	0.7 (0.4–1.0)	—	**0.8 (0.6–1.0)**	**200,000**
Heated tobacco products	0.6 (0.4–0.8)	0.7 (0.5–1.0)	0.6 (0.5–0.9)	0.7 (0.4–1.3)	0.7 (0.4–1.1)	—	**0.7 (0.5–0.8)**	**170,000**
Pipe tobacco	0.3 (0.2–0.5)	0.3 (0.2–0.6)	0.4 (0.2–0.6)	—	0.3 (0.2–0.6)	—	**0.3 (0.2–0.5)**	**80,000**
Any combustible tobacco product^††^	3.1 (2.6–3.8)	3.2 (2.8–3.7)	3.1 (2.6–3.7)	5.2 (4.1–6.6)	2.8 (2.3–3.4)	—	**3.2 (2.8–3.6)**	**860,000**
Two or more tobacco products^§§^	2.4 (1.9–2.9)	3.0 (2.5–3.7)	3.1 (2.6–3.8)	3.0 (2.2–4.0)	2.2 (1.7–2.9)	—	**2.7 (2.3–3.2)**	**740,000**
**High school (grades 9–12)**								
**Any tobacco product**	**13.8 (11.9–16.0)**	**13.0 (11.2–15.1)**	**16.2 (14.1–18.6)**	**11.0 (8.7–13.9)**	**9.1 (7.4–11.1)**	**9.3 (6.3–13.6)**	**13.4 (11.8–15.2)**	**2,060,000**
E-cigarettes	11.9 (10.0–14.0)	10.7 (9.1–12.6)	14.5 (12.4–16.8)	5.9 (4.2–8.2)	7.6 (6.0–9.6)	7.4 (4.9–10.9)	**11.3 (9.7–13.0)**	**1,720,000**
Cigarettes	1.8 (1.3–2.3)	2.0 (1.5–2.7)	2.2 (1.8–2.8)	—	1.6 (1.2–2.3)	—	**1.9 (1.5–2.4)**	**280,000**
Cigars	1.5 (1.1–2.1)	2.6 (2.1–3.3)	2.1 (1.6–2.6)	4.4 (3.3–5.9)	1.2 (0.8–1.6)	—	**2.1 (1.7–2.6)**	**310,000**
Smokeless tobacco	—	1.7 (1.2–2.4)	1.5 (1.0–2.1)	—	—	—	**1.2 (0.8–1.6)**	**170,000**
Hookahs	1.3 (0.8–2.0)	1.2 (0.9–1.5)	0.8 (0.5–1.1)	3.2 (1.9–5.2)	1.3 (0.8–2.3)	—	**1.2 (0.9–1.6)**	**180,000**
Nicotine pouches	0.6 (0.3–1.0)	1.6 (1.1–2.3)	1.4 (0.9–2.2)	—	—	—	**1.1 (0.8–1.6)**	**160,000**
Heated tobacco products	0.7 (0.5–1.2)	0.9 (0.6–1.4)	0.9 (0.6–1.3)	—	—	—	**0.8 (0.6–1.1)**	**120,000**
Pipe tobacco	—	—	0.5 (0.3–0.9)	—	—	—	**0.4 (0.3–0.7)**	**60,000**
Any combustible tobacco product	4.2 (3.3–5.2)	4.6 (3.8–5.4)	4.3 (3.5–5.1)	7.3 (5.5–9.6)	3.5 (2.7–4.5)	—	**4.4 (3.8–5.1)**	**660,000**
Two or more tobacco products	3.1 (2.4–4.0)	4.5 (3.6–5.6)	4.4 (3.7–5.4)	4.0 (2.8–5.7)	2.6 (2.0–3.5)	—	**3.8 (3.2–4.6)**	**580,000**
**Middle school (grades 6–8)**								
**Any tobacco product**	**4.4 (3.5–5.5)**	**3.6 (2.9–4.5)**	**3.4 (2.7–4.4)**	**4.5 (3.4–5.9)**	**5.3 (3.9–7.1)**	**2.2 (1.2–3.8)**	**4.0 (3.3–4.8)**	**470,000**
E-cigarettes	3.2 (2.4–4.2)	2.3 (1.9–3.0)	2.6 (1.9–3.5)	2.3 (1.4–4.0)	3.9 (2.9–5.3)	—	**2.8 (2.2–3.4)**	**320,000**
Cigarettes	1.2 (0.8–1.8)	0.9 (0.6–1.4)	1.0 (0.7–1.5)	1.1 (0.6–1.9)	1.2 (0.7–1.9)	—	**1.0 (0.8–1.4)**	**120,000**
Cigars	0.5 (0.3–0.9)	0.6 (0.4–0.9)	0.5 (0.3–0.7)	1.4 (0.8–2.3)	0.6 (0.4–1.1)	—	**0.6 (0.4–0.8)**	**60,000**
Smokeless tobacco	—	0.8 (0.5–1.2)	0.5 (0.3–0.9)	—	—	—	**0.6 (0.4–0.9)**	**60,000**
Hookahs	—	—	—	—	—	—	**0.4 (0.2–0.6)**	**40,000**
Nicotine pouches	—	0.2 (0.1–0.3)	—	—	—	—	**0.3 (0.2–0.5)**	**30,000**
Heated tobacco products	0.4 (0.2–0.7)	0.4 (0.3–0.7)	—	—	—	—	**0.4 (0.3–0.6)**	**40,000**
Pipe tobacco	—	—	—	—	—	—	**0.2 (0.1–0.3)**	**20,000**
Any combustible tobacco product	1.9 (1.3–2.7)	1.4 (1.0–1.9)	1.4 (1.0–1.9)	2.4 (1.7–3.3)	1.9 (1.3–2.8)	—	**1.6 (1.3–2.1)**	**190,000**
Two or more tobacco products	1.5 (0.9–2.3)	1.1 (0.8–1.5)	1.2 (0.8–1.8)	1.6 (1.0–2.5)	1.6 (1.0–2.7)	—	**1.3 (0.9–1.7)**	**150,000**

**Abbreviation: **e-cigarettes = electronic cigarettes.

* Past 30-day use of e-cigarettes was determined by asking, “During the past 30 days, on how many days did you use e-cigarettes?” Past 30-day use of cigarettes was determined by asking, “During the past 30 days, on how many days did you smoke cigarettes?” Past 30-day use of cigars was determined by asking, “During the past 30 days, on how many days did you smoke cigars, cigarillos, or little cigars?” Smokeless tobacco was defined as use of chewing tobacco, snuff, or dip, snus, or dissolvable tobacco products. Past 30-day use of smokeless tobacco was determined by asking, “During the past 30 days, on how many days did you use [chewing tobacco, snuff, or dip, snus, or dissolvable tobacco products]?” Responses from these questions were combined to derive overall smokeless tobacco use. Past 30-day use of hookahs was determined by asking, “During the past 30 days, on how many days did you smoke tobacco in a hookah or water pipe?” Past 30-day use of nicotine pouches was determined by asking, “During the past 30 days, on how many days did you use nicotine pouches?” Past 30-day use of heated tobacco products was determined by asking, “During the past 30 days, on how many days did you use heated tobacco products?” Past 30-day use of pipe tobacco (not hookahs) was determined by asking, “In the past 30 days, on how many days did you smoke pipes filled with tobacco?” Because of missing data on the past 30-day use questions, denominators for each tobacco product might be different. For each product, current use was defined as self-reported use on ≥1 day during the past 30 days.

† Hispanic persons could be of any race.

§ Estimated weighted total number of current tobacco product users was rounded down to the nearest 10,000 persons. Overall estimates were reported among 20,413 U.S. middle and high school students. School level was determined by self-reported grade level: high school (grades 9–12; n = 10,515) and middle school (grades 6–8; n = 9,763). Overall estimates might not directly total to sums of corresponding subgroup estimates because of rounding or inclusion of students who did not self-report sex, race and ethnicity, or grade level.

¶ Any tobacco product use was defined as use of any tobacco product (e-cigarettes, cigarettes, cigars [cigars, cigarillos, or little cigars], smokeless tobacco [chewing tobacco, snuff, or dip, snus, or dissolvable tobacco products], hookahs, pipe tobacco, heated tobacco products, nicotine pouches, or bidis [small brown cigarettes wrapped in a leaf]) on ≥1 day during the past 30 days.

** Data were statistically unreliable because of unweighted denominator <50 or a relative standard error >30%.

†† Any combustible tobacco product use was defined as use of cigarettes, cigars (cigars, cigarillos, or little cigars), hookahs, pipe tobacco, or bidis on ≥1 day during the past 30 days.

§§ Defined as use of two or more tobacco products (e-cigarettes, cigarettes, cigars [cigars, cigarillos, or little cigars], smokeless tobacco [chewing tobacco, snuff, or dip, snus, or dissolvable tobacco products], hookahs, pipe tobacco, heated tobacco products, nicotine pouches, or bidis) on ≥1 day during the past 30 days.

**FIGURE 1:**
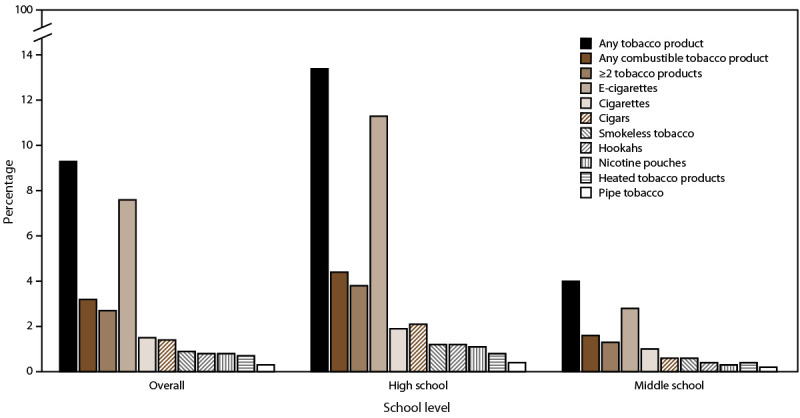
Percentage of middle and high school students who reported current (past 30-day) use of select tobacco products,* any tobacco product,^†^ any combustible tobacco product,^§^ or two or more tobacco product types,^¶^ by school level** and overall — National Youth Tobacco Survey, United States, 2021 **Abbreviation:** e-cigarettes = electronic cigarettes. * Past 30-day use of e-cigarettes was determined by asking, “During the past 30 days, on how many days did you use e-cigarettes?” Past 30-day use of cigarettes was determined by asking, “During the past 30 days, on how many days did you smoke cigarettes?” Past 30-day use of cigars was determined by asking, “During the past 30 days, on how many days did you smoke cigars, cigarillos, or little cigars?” Smokeless tobacco was defined as use of chewing tobacco, snuff, dip, snus, or dissolvable tobacco products. Past 30-day use of smokeless tobacco was determined by asking, “During the past 30 days, on how many days did you use [chewing tobacco, snuff, or dip /snus/dissolvable tobacco products]?” Responses from these questions were combined to derive overall smokeless tobacco use. Past 30-day use of hookahs was determined by asking, “During the past 30 days, on how many days did you smoke tobacco in a hookah or water pipe?” Past 30-day use of nicotine pouches was determined by asking, “During the past 30 days, on how many days did you use nicotine pouches?” Past 30-day use of heated tobacco products was determined by asking, “During the past 30 days, on how many days did you use heated tobacco products?” Past 30-day use of pipe tobacco (not hookahs) was determined by asking, “In the past 30 days, on how many days did you smoke pipes filled with tobacco?” Because of missing data on the past 30-day use questions, denominators for each tobacco product might be different. For each product, current use was defined as self-reported use on ≥1 day during the past 30 days. ^†^ Any tobacco product use was defined as use of any tobacco product (e-cigarettes, cigarettes, cigars [cigars, cigarillos, or little cigars], smokeless tobacco [chewing tobacco, snuff, dip, snus, or dissolvable tobacco products], hookahs, pipe tobacco, heated tobacco products, nicotine pouches, or bidis [small brown cigarettes wrapped in a leaf]) on ≥1 day during the past 30 days. ^§^ Any combustible tobacco product use was defined as use of cigarettes, cigars (cigars, cigarillos, or little cigars), hookahs, pipe tobacco, or bidis on ≥1 day during the past 30 days. ^¶^ Defined as use of two or more tobacco products (e-cigarettes, cigarettes, cigars [cigars, cigarillos, or little cigars], smokeless tobacco [chewing tobacco, snuff, dip, snus, or dissolvable tobacco products], hookahs, pipe tobacco, heated tobacco products, nicotine pouches, or bidis) on ≥1 day during the past 30 days. ** Overall estimates were reported among 20,413 U.S. middle and high school students. School level was determined by self-reported grade level: high school (grades 9–12; n = 10,515) and middle school (grades 6–8; n = 9,763).

Among high school students, 13.4% (2.06 million) reported current use of any tobacco product (Figure 1). Among high school students who currently used tobacco products, 32.8% (660,000) currently used any combustible tobacco product and 28.4% (580,000) currently used two or more tobacco product types. Among high school students, e-cigarettes were the most commonly used tobacco product (11.3%; 1.72 million), followed by cigars (2.1%), cigarettes (1.9%), hookahs and smokeless tobacco (both 1.2%), nicotine pouches (1.1%), HTPs (0.8%), and pipe tobacco (0.4%).

Among middle school students, 4.0% (470,000) reported current use of any tobacco product. Among middle school students who currently used tobacco products, 40.0% (190,000) currently used any combustible tobacco product and 32.5% (150,000) currently used two or more tobacco product types. Among middle school students, e-cigarettes were the most commonly used tobacco product (2.8%; 320,000), followed by cigarettes (1.0%), cigars and smokeless tobacco (both 0.6%), hookahs and HTPs (both 0.4%), nicotine pouches (0.3%), and pipe tobacco (0.2%).

### Social Determinant Indicators

#### Ever Tobacco Product Use

By race and ethnicity, ever use of any tobacco product was reported by 26.7% of students who were non-Hispanic White, 22.4% of students who were Hispanic, 21.9% of students who were non-Hispanic Black, and 16.1% of students who were non-Hispanic other race (Table 1). Ever use of any combustible tobacco product was reported by 14.0% of students who were non-Hispanic Black, 12.6% of students who were non-Hispanic White, 10.4% of students who were Hispanic, and 8.3% of students who were non-Hispanic other race.

Ever use of any tobacco product was reported by 35.4% of students identifying as LGB, 22.8% of those identifying as heterosexual, and 14.4% of those who were not sure (Table 3) and 37.9% of those identifying as transgender, 23.4% of those identifying as not transgender, and 26.4% of those who were not sure.

**TABLE 3:** Percentage of middle and high school students who reported ever use or current (past 30-day) use of any tobacco product,* overall and by selected demographic characteristics and social determinant indicators — National Youth Tobacco Survey, United States, 2021

Characteristic	Ever use, any tobacco product		Current use, any tobacco product	
	% (95% CI)	Estimated no. of users^†^	% (95% CI)	Estimated no. of users^†^
**Overall^§^**				
**Sexual identity**				
Heterosexual	22.8 (20.7–25.0)	4,210,000	7.9 (7.0–9.0)	1,460,000
Gay, lesbian, or bisexual	35.4 (32.4–38.4)	1,220,000	14.2 (11.9–17.0)	490,000
Not sure	14.4 (11.8–17.5)	340,000	5.5 (4.2–7.3)	130,000
**Transgender**				
No, not transgender	23.4 (21.3–25.7)	5,210,000	8.2 (7.3–9.3)	1,830,000
Yes, transgender	37.9 (31.3–45.0)	160,000	18.9 (13.8–25.4)	80,000
Not sure	26.4 (20.7–33.1)	180,000	9.1 (6.1–13.5)	60,000
I don’t know what this question is asking	18.4 (14.7–22.8)	180,000	9.7 (6.9–13.4)	90,000
**Psychological distress (PHQ-4 scale)^¶^**				
None	16.6 (14.7–18.8)	2,070,000	5.5 (4.7–6.3)	680,000
Mild	27.3 (25.0–29.7)	1,340,000	9.6 (8.3–11.1)	470,000
Moderate	29.3 (25.7–33.2)	910,000	11.2 (8.8–14.1)	340,000
Severe	37.8 (33.9–41.8)	1,110,000	14.2 (11.9–16.8)	410,000
Family affluence scale**				
Low	24.4 (22.4–26.6)	1,490,000	9.2 (7.8–10.8)	560,000
Medium	22.3 (20.2–24.6)	2,400,000	7.7 (6.7–8.8)	820,000
High	24.0 (20.9–27.5)	1,760,000	8.8 (7.5–10.3)	640,000
**Grades in school**				
Mostly As	18.3 (15.9–21.0)	1,980,000	5.5 (4.6–6.7)	590,000
Mostly Bs	25.7 (23.3–28.3)	1,770,000	9.6 (8.2–11.1)	650,000
Mostly Cs	31.3 (28.3–34.4)	910,000	13.2 (11.0–15.8)	380,000
Mostly Ds	36.7 (32.1–41.6)	360,000	15.6 (12.6–19.2)	150,000
Mostly Fs	41.7 (36.8–46.8)	350,000	17.3 (13.4–22.0)	140,000
**Speak language other than English (home)**				
Yes	20.5 (18.4–22.8)	1,440,000	6.4 (5.5–7.3)	440,000
No	24.5 (22.1–27.1)	4,310,000	9.2 (8.1–10.5)	1,620,000
**High school (grades 9–12)**				
**Sexual identity**				
Heterosexual	31.6 (29.2–34.1)	3,420,000	11.4 (10.1–12.9)	1,230,000
Gay, lesbian, or bisexual	43.8 (40.1–47.6)	910,000	17.4 (14.2–21.1)	360,000
Not sure	26.8 (22.5–31.7)	240,000	10.2 (7.3–13.9)	90,000
**Transgender**				
No, not transgender	32.7 (30.3–35.2)	4,190,000	11.7 (10.3–13.2)	1,490,000
Yes, transgender	45.6 (36.7–54.8)	110,000	24.5 (17.2–33.6)	60,000
Not sure	38.4 (29.3–48.3)	120,000	12.3 (6.8–21.4)	30,000
I don’t know what this question is asking	30.9 (24.0–38.9)	140,000	17.9 (12.1–25.5)	80,000
**Psychological distress (PHQ-4 scale)**				
None	25.4 (22.9–28.0)	1,680,000	8.3 (7.2–9.6)	550,000
Mild	37.1 (33.9–40.3)	1,100,000	13.6 (11.5–16.0)	400,000
Moderate	37.5 (32.6–42.6)	710,000	15.2 (11.7–19.5)	290,000
Severe	46.5 (42.5–50.5)	840,000	17.6 (14.5–21.2)	310,000
**Family affluence scale**				
Low	33.2 (30.5–36.0)	1,170,000	12.7 (10.4–15.4)	440,000
Medium	31.4 (28.5–34.5)	1,930,000	11.2 (9.6–13.0)	680,000
High	34.9 (31.7–38.3)	1,400,000	12.8 (11.0–14.9)	510,000
**Grades in school**				
Mostly As	27.8 (24.6–31.2)	1,680,000	8.5 (7.1–10.2)	510,000
Mostly Bs	35.4 (32.6–38.2)	1,420,000	13.7 (11.8–15.8)	550,000
Mostly Cs	40.7 (36.8–44.7)	710,000	17.9 (14.8–21.5)	310,000
Mostly Ds	42.6 (35.2–50.4)	250,000	18.4 (13.1–25.2)	100,000
Mostly Fs	48.3 (41.8–54.9)	260,000	19.6 (14.4–26.0)	100,000
**Speak language other than English (home)**				
Yes	28.5 (25.7–31.4)	1,070,000	8.4 (6.7–10.4)	310,000
No	34.5 (32.0–37.1)	3,480,000	13.5 (11.9–15.2)	1,350,000
**Middle school (grades 6–8)**				
**Sexual identity**				
Heterosexual	10.0 (8.7–11.6)	760,000	3.0 (2.4–3.6)	220,000
Gay, lesbian, or bisexual	22.5 (18.8–26.6)	300,000	9.5 (7.1–12.5)	120,000
Not sure	6.8 (4.6–9.9)	100,000	2.6 (1.6–4.0)	30,000
**Transgender**				
No, not transgender	10.6 (9.2–12.2)	990,000	3.5 (2.9–4.3)	330,000
Yes, transgender	27.8 (19.0–38.7)	50,000	—^††^	—
Not sure	16.7 (11.6–23.4)	60,000	6.5 (3.7–11.0)	20,000
I don’t know what this question is asking	7.4 (5.0–10.9)	40,000	2.3 (1.4–3.9)	10,000
**Psychological distress (PHQ-4 scale)**				
None	6.3 (5.3–7.5)	360,000	2.1 (1.6–2.7)	120,000
Mild	12.2 (10.0–14.8)	230,000	3.4 (2.5–4.5)	60,000
Moderate	16.4 (13.1–20.2)	190,000	4.9 (3.3–7.1)	50,000
Severe	24.1 (19.6–29.3)	270,000	8.8 (6.3–12.1)	90,000
**Family affluence scale**				
Low	12.3 (10.4–14.5)	310,000	4.4 (3.6–5.4)	110,000
Medium	10.0 (8.3–11.9)	450,000	3.0 (2.2–4.0)	130,000
High	10.8 (8.8–13.1)	350,000	3.8 (2.9–4.9)	120,000
**Grades in school**				
Mostly As	6.0 (4.8–7.3)	280,000	1.7 (1.2–2.4)	70,000
Mostly Bs	12.2 (10.0–14.9)	340,000	3.7 (2.7–5.0)	100,000
Mostly Cs	17.0 (13.9–20.7)	190,000	6.1 (4.5–8.3)	70,000
Mostly Ds	27.9 (21.4–35.5)	110,000	11.2 (7.6–16.1)	40,000
Mostly Fs	30.4 (22.0–40.4)	90,000	13.3 (9.1–19.1)	40,000
**Speak language other than English (home)**				
Yes	11.1 (8.8–13.9)	350,000	3.8 (2.8–5.2)	120,000
No	10.9 (9.2–12.9)	810,000	3.6 (2.8–4.5)	260,000

**Abbreviations: **e-cigarettes = electronic cigarettes; PHQ-4 = four-item Patient Health Questionnaire.

* Any tobacco product includes e-cigarettes, cigarettes, cigars (cigars, cigarillos, or little cigars), smokeless tobacco (chewing tobacco, snuff, or dip; snus; dissolvable tobacco products), hookahs, pipe tobacco, bidis (small brown cigarettes wrapped in a leaf), heated tobacco products and nicotine pouches. Ever use of any tobacco product was defined as self-reported ever use of any tobacco product, even just one time. Current use of any tobacco product was defined as self-reported use of any tobacco product on ≥1 day during the past 30 days.

^† ^Estimated weighted total numbers of ever and current tobacco product users were rounded down to the nearest 10,000 persons. Overall estimates might not directly total to sums of corresponding subgroup estimates by school level because of rounding or inclusion of students who did not self-report grade level.

^§ ^Overall estimates were reported among 20,413 U.S. middle and high school students. School level was determined by self-reported grade level: high school (grades 9–12; n = 10,515) and middle school (grades 6–8; n = 9,763).

^¶ ^A composite scale made up of four questions to assess psychological distress: “During the past two weeks, how often have you been bothered by any of the following problems?”: 1) little interest or pleasure in doing things; 2) feeling down, depressed, or hopeless; 3) feeling nervous, anxious, or on edge; 4) not being able or stop or control worrying. Response options were provided with a numeric value [not at all = 0; several days = 1; more than half of the days = 2; and nearly every day = 3]. Responses were summed (range: 0–12) and categorized as none (0–2), mild (3–5), moderate (6–8) and severe (9–12). The PHQ–4 scale was reported among respondents who had complete data on all four questions (n = 17,355).

** A composite scale made up of four questions. Numeric values were assigned to each response and summed across responses: 1) “Does your family own a vehicle (such as a car, van, or truck)?” [no = 0; yes, one = 1; yes, two or more = 2]; 2) “Do you have your own bedroom?” [no = 0; yes = 1]; 3) “How many computers (including laptops and tablets, not including game consoles and smartphones) does your family own?” [none = 0; one= 1; two = 2; more than two = 3]; and 4) “During the past 12 months, how many times did you travel on vacation with your family?” [not at all = 0; once = 1; twice = 2; more than twice = 3]. Summed responses (range: 0–9) were categorized into approximate tertiles based on the weighted distribution of scores in this sample: low (0–5), medium (6–7), and high (8–9). The family affluence scale was reported among respondents who had complete data on all four questions (n = 17,878).

**^†† ^**Data were statistically unreliable because of unweighted denominator <50 or a relative standard error >30%.

For psychological distress, prevalence of ever use of any tobacco product ranged from 16.6% (none) to 37.8% (severe) (Table 3). Prevalence was similar for low (24.4%), medium (22.3%), and high (24.0%) family affluence. Prevalence of ever use of any tobacco product increased as self-reported grades in school declined (mostly As: 18.3% to mostly Fs: 41.7%). Ever use was 24.5% for students who spoke English at home and 20.5% for those who spoke another language at home.

#### Current Tobacco Product Use

By race and ethnicity, current use of any tobacco product was reported by 11.0% of students who were non-Hispanic White, 8.2% of students who were non-Hispanic Black, 7.4% of students who were Hispanic, and 5.4% of students who were non-Hispanic other race (Table 2). Current use of any combustible tobacco product was reported by 5.2% of students who were non-Hispanic Black, 3.1% of students who were non-Hispanic White, and 2.8% of students who were Hispanic.

Overall, current use of any tobacco product was reported by 14.2% of students identifying as LGB, 7.9% of those identifying as heterosexual, and 5.5% of those who were not sure (Table 3) and 18.9% of those identifying as transgender, 8.2% of those identifying as not transgender, and 9.1% of those who were not sure.

By psychological distress, prevalence of current use of any tobacco product ranged from 5.5% (none) to 14.2% (severe) (Table 3). Current use was similar for low (9.2%), medium (7.7%), and high (8.8%) family affluence. Prevalence of current use of any tobacco product increased with declining self-reported grades in school (mostly As: 5.5% to mostly Fs: 17.3%). Current use of any tobacco product was 9.2% for students who spoke English in the home and 6.4% for those spoke another language at home.

### Frequency of Tobacco Product Use

Among students who currently used each tobacco product, frequent use (on ≥20 of the past 30 days) was 39.4% (810,000) for e-cigarettes, 29.5% (50,000) for chewing tobacco, snuff, or dip, 26.1% (20,000) for snus, 22.1% (30,000) for HTPs, 20.7% (80,000) for cigars, 19.9% (40,000) for hookahs, 18.9% (70,000) for cigarettes, and 17.2% (30,000) for nicotine pouches (Table 4). For nearly all tobacco products, more than half of students who currently used the products reported using them on 1–5 days of the past 30 days.

**TABLE 4:** Frequency of use* among middle and high school students currently (past 30-day) using each tobacco product,^†^ overall and by school level — National Youth Tobacco Survey, United States, 2021

Characteristic	1–5 days		6–19 days		20–30 days	
	% (95% CI)	Estimated no. of users^§^	% (95% CI)	Estimated no. of users^§^	% (95% CI)	Estimated no. of users^§^
**Overall**						
E-cigarettes	44.5 (40.7–48.3)	910,000	16.2 (13.8–18.8)	330,000	39.4 (35.4–43.5)	810,000
Cigarettes	66.5 (57.8–74.3)	270,000	14.6 (10.4–20.1)	60,000	18.9 (13.6–25.7)	70,000
Cigars	67.1 (60.0–73.5)	250,000	12.2 (8.3–17.7)	40,000	20.7 (15.3–27.4)	80,000
Chewing tobacco, snuff, or dip	50.5 (40.1–60.9)	100,000	20.0 (13.8–28.0)	30,000	29.5 (22.4–37.9)	50,000
Snus	57.3 (41.7–71.6)	50,000	—^¶^	—	26.1 (15.5–40.6)	20,000
Hookahs	66.2 (57.1–74.3)	140,000	13.9 (9.0–20.9)	30,000	19.9 (14.0–27.5)	40,000
Pipe tobacco	65.3 (45.8–80.7)	50,000	—	—	—	—
Heated tobacco products	54.3 (42.9–65.3)	90,000	23.6 (15.3–34.7)	40,000	22.1 (13.7–33.5)	30,000
Nicotine pouches	63.5 (53.5–72.5)	120,000	19.3 (11.7–30.1)	30,000	17.2 (11.3–25.3)	30,000
**High school (grades 9–12)**						
E-cigarettes	40.2 (36.0–44.5)	690,000	16.3 (13.5–19.5)	280,000	43.6 (39.0–48.2)	750,000
Cigarettes	66.9 (56.5–75.9)	190,000	13.3 (8.7–19.7)	30,000	19.9 (13.8–27.6)	50,000
Cigars	67.3 (59.0–74.6)	210,000	12.0 (7.7–18.3)	30,000	20.7 (14.6–28.5)	60,000
Chewing tobacco, snuff, or dip	47.6 (34.8–60.6)	60,000	22.2 (14.2–33.0)	30,000	30.3 (21.8–40.2)	40,000
Snus	—	—	—	—	—	—
Hookahs	68.2 (57.5–77.3)	120,000	14.7 (9.3–22.6)	20,000	17.0 (11.0–25.5)	30,000
Pipe tobacco	—	—	—	—	—	—
Heated tobacco products	55.6 (41.7–68.7)	60,000	19.7 (12.2–30.3)	20,000	24.7 (13.6–40.6)	30,000
Nicotine pouches	64.1 (52.1–74.6)	100,000	21.0 (12.2–33.5)	30,000	15.0 (9.1–23.7)	20,000
**Middle school (grades 6–8)**						
E-cigarettes	66.7 (60.0–72.8)	210,000	16.1 (11.3–22.6)	50,000	17.2 (12.8–22.6)	50,000
Cigarettes	68.0 (56.1–77.9)	80,000	14.8 (8.2–25.1)	10,000	17.2 (9.3–29.8)	20,000
Cigars	66.7 (51.3–79.1)	40,000	—	—	20.1 (10.8–34.4)	10,000
Chewing tobacco, snuff, or dip	57.6 (43.2–70.7)	30,000	—	—	28.0 (16.0–44.3)	10,000
Snus	—	—	—	—	—	—
Hookahs	—	—	—	—	—	—
Pipe tobacco	—	—	—	—	—	—
Heated tobacco products	51.0 (30.1–71.5)	20,000	—	—	—	—
Nicotine pouches	—	—	—	—	—	—

**Abbreviation: **e-cigarettes = electronic cigarettes.

* Frequency of current use of e-cigarettes; cigarettes; cigars (cigars, cigarillos, or little cigars); chewing tobacco, snuff, or dip; snus; dissolvable tobacco products; pipe tobacco; hookahs; heated tobacco products; and nicotine pouches was determined by asking respondents on how many days they smoked or used each respective tobacco product during the past 30 days. Respondents could enter a valid response of 0–30 days. Responses were recoded as 1–5 days, 6–19 days, and 20–30 days.

^† ^Estimates of frequency of use of dissolvable tobacco products are statistically unreliable (overall and by school level) and are omitted from the table.

^§^ Estimated weighted total number of users was rounded down to the nearest 10,000 persons. Overall estimates might not directly total to sums of corresponding subgroup estimates because of rounding or inclusion of students who did not self-report grade level.

^¶^ Data were statistically unreliable because of unweighted denominator <50 or a relative standard error >30%.

### Flavored Tobacco Product Use

Among students who currently used any tobacco product, 79.1% (high school: 80.2%; middle school: 74.6%) reported using flavored tobacco product(s) in the past 30 days (Table 5). Among students who currently used each tobacco product, use of a flavored product was 84.7% for e-cigarettes, 70.1% for smokeless tobacco, 61.6% for nicotine pouches, 46.6% for hookahs, 44.4% for cigars, 44.0% for HTPs, 38.8% for cigarettes (menthol only), and 34.4% for pipe tobacco.

**TABLE 5:** Flavored tobacco product* use among middle and high school students overall and among those who reported current (past 30-day) use^†^ of each tobacco product, by school level, sex, and race and ethnicity — National Youth Tobacco Survey, United States, 2021

Characteristic	Tobacco product								
	Any tobacco^§^	E-cigarettes	Cigarettes^¶^	Cigars	Smokeless tobacco**	Hookahs	Pipe tobacco	Heated tobacco products	Nicotine pouches
	% (95% CI)	% (95% CI)	% (95% CI)	% (95% CI)	% (95% CI)	% (95% CI)	% (95% CI)	% (95% CI)	% (95% CI)
**Overall**									
**Flavored tobacco product use among all students^††^**	**7.2 (6.2–8.2)**	**6.2 (5.3–7.3)**	**0.6 (0.4–0.8)**	**0.6 (0.5–0.8)**	**0.6 (0.5–0.9)**	**0.4 (0.3–0.5)**	**0.1 (0.1–0.2)**	**0.3 (0.2–0.4)**	**0.5 (0.3–0.7)**
**Current use**									
Estimated weighted no. of flavored tobacco product users^§§^	1,950,000	1,680,000	150,000	160,000	170,000	100,000	30,000	70,000	120,000
Flavored tobacco product use among current tobacco users^¶¶^	79.1 (76.0–81.9)	84.7 (81.4–87.5)	38.8 (32.3–45.7)	44.4 (36.9–52.1)	70.1 (60.8–77.9)	46.6 (38.3–55.2)	34.4 (20.1–52.1)	44.0 (34.0–54.5)	61.6 (49.7–72.2)
**School level**									
High school (grades 9–12)	80.2 (76.8–83.3)	85.8 (82.3–88.7)	41.1 (33.2–49.5)	41.1 (32.9–49.7)	72.1 (60.3–81.5)	42.9 (33.1–53.3)	—***	41.0 (27.8–55.7)	63.9 (50.1–75.7)
Middle school (grades 6–8)	74.6 (67.5–80.6)	79.2 (69.1–86.6)	34.8 (24.9–46.3)	59.9 (46.5–72.0)	65.5 (50.9–77.6)	—	—	51.5 (35.2–67.4)	—
**Sex**									
Female	84.3 (80.2–87.7)	88.8 (85.1–91.6)	41.8 (32.5–51.8)	55.8 (43.3–67.6)	61.9 (44.3–76.8)	52.2 (39.5–64.6)	—	55.1 (37.6–71.4)	—
Male	74.1 (70.0–77.8)	80.5 (75.7–84.6)	37.0 (28.5–46.4)	38.2 (29.9–47.3)	72.6 (61.0–81.8)	40.4 (29.5–52.4)	—	35.5 (21.1–53.1)	60.3 (45.0–73.8)
**Race and ethnicity**									
White, non-Hispanic	82.9 (79.4–85.9)	86.5 (82.5–89.7)	38.2 (29.4–47.7)	43.6 (34.8–52.8)	75.7 (62.9–85.2)	36.0 (21.6–53.4)	—	37.7 (23.8–54.0)	65.2 (48.7–78.7)
Black, non-Hispanic	66.2 (58.8–72.8)	76.3 (61.8–86.6)	—	46.3 (33.6–59.4)	—	50.4 (36.0–64.8)	—	—	—
Hispanic^†††^	76.4 (71.2–80.9)	83.6 (78.0–88.0)	28.6 (19.4–40.1)	45.3 (29.0–62.7)	—	—	—	—	—
Other, non-Hispanic	79.4 (67.1–87.9)	86.6 (72.3–94.2)	—	—	—	—	—	—	—

**Abbreviation: **e-cigarettes = electronic cigarettes.

* For each respective tobacco product excluding cigarettes, current (past 30-day) users were asked, “Were any of the [tobacco product] that you used in the past 30 days flavored to taste like menthol, mint, clove or spice, alcohol (wine, cognac), candy, fruit, chocolate, or any other flavor?” Response options were “yes,” “no,” or “don’t know.” Those who responded yes were considered flavored tobacco product users.

^†^ Reported among respective current (past 30-day) users for each product. Past 30-day use of e-cigarettes was determined by asking, “During the past 30 days, on how many days did you use e-cigarettes?” Past 30-day use of cigarettes was determined by asking, “During the past 30 days, on how many days did you smoke cigarettes?” Past 30-day use of cigars was determined by asking, “During the past 30 days, on how many days did you smoke cigars, cigarillos, or little cigars?” Past 30-day use of smokeless tobacco was determined by asking the following question for use of chewing tobacco, snuff, and dip: “During the past 30 days, on how many days did you use chewing tobacco, snuff, or dip?” Past 30-day use of snus was determined by asking the following question for use of snus: “During the past 30 days, on how many days did you use snus?” Past 30-day use of dissolvable tobacco products was determined by asking the following question for use of dissolvable tobacco products: “During the past 30 days, on how many days did you use dissolvable tobacco products?” Past 30-day use of pipe tobacco was determined by asking the following question for use of pipe tobacco: “During the past 30 days, on how many days did you smoke pipes filled with tobacco?” Past 30-day use of hookahs was determined by asking, “During the past 30 days, on how many days did you smoke tobacco in a hookah or water pipe?” Past 30-day use of heated tobacco products was determined by asking the following question for use of heated tobacco products: “During the past 30 days, on how many days did you use heated tobacco products?” Past 30-day use of nicotine pouches was determined by asking the following question for use of heated tobacco products: “During the past 30 days, on how many days did you use nicotine pouches?”

^§^ Any tobacco use includes use of e-cigarettes, cigarettes, cigars (cigars, cigarillos, or little cigars), smokeless tobacco (chewing tobacco, snuff, or dip; snus; dissolvable tobacco products), hookahs, pipe tobacco, bidis (small brown cigarettes wrapped in a leaf), heated tobacco products, and nicotine pouches on ≥1 day during the past 30 days.

^¶^ Flavored cigarette use refers to menthol cigarette use. Current cigarette smokers were categorized as flavored (menthol) cigarette smokers: if they responded “yes” to the question, “Menthol cigarettes are cigarettes that taste like mint. During the past 30 days, were the cigarettes that you usually smoked menthol?”; or if they indicated “Kool” or “Newport” as a brand they usually smoked in the past 30 days. Usual brand was determined based on responses to two questions: 1) “During the past 30 days, what brands of cigarettes did you smoke? (Select one or more)” and 2) “During the past 30 days, what brand of cigarettes did you usually smoke? (Choose only one answer).” If “Kool” or “Newport” was the only brand selected for the first question, or if multiple brands were selected in the first question and “Kool” or “Newport” was selected for the second question, “Kool” or “Newport” was considered the respondent’s usual brand. Those who selected “some other brand(s) not listed here” could provide a write-in response; write-in responses corresponding to an original response option were recoded. Those who reported “no” or “not sure” to the menthol question or those who did not report “Newport” or “Kool” as their usual brand were categorized as nonmenthol smokers; all other past 30-day cigarette smokers who did not provide any valid response were assigned as missing menthol smoking status.

** Flavored tobacco product use was assessed separately among current (past 30-day) users of chewing tobacco, snuff, or dip; snus; and dissolvable tobacco product users. Flavored use was then recoded as a composite among users of any current smokeless tobacco product combined.

^††^ Calculated among all respondents regardless of tobacco product use status. Because of missing data, denominators for each tobacco product might be different: any tobacco (n = 20,167), e-cigarettes (n = 20,084), cigarettes (n = 19,936), cigars (n = 19,860), smokeless tobacco [chewing tobacco, snuff, or dip; snus; dissolvable tobacco products] (n = 19,778), hookahs (n = 19,697), pipe tobacco (n = 19,655), heated tobacco products (n = 19,501), and nicotine pouches (n = 19,500).

^§§^ Estimated weighted total number of flavored tobacco product users was rounded down to the nearest 10,000 persons.

^¶¶^ Calculated among current users of any tobacco (n =1,792), e-cigarettes (n = 1,383), cigarettes (n = 314), cigars (n = 299), smokeless tobacco (n = 210), hookahs (n = 168), pipe tobacco (n = 64), heated tobacco products (n = 133), and nicotine pouches (n = 137).

*** Data were statistically unreliable because of unweighted denominator <50 or a relative standard error >30%.

^†††^ Hispanic persons could be of any race.

**FIGURE 2:**
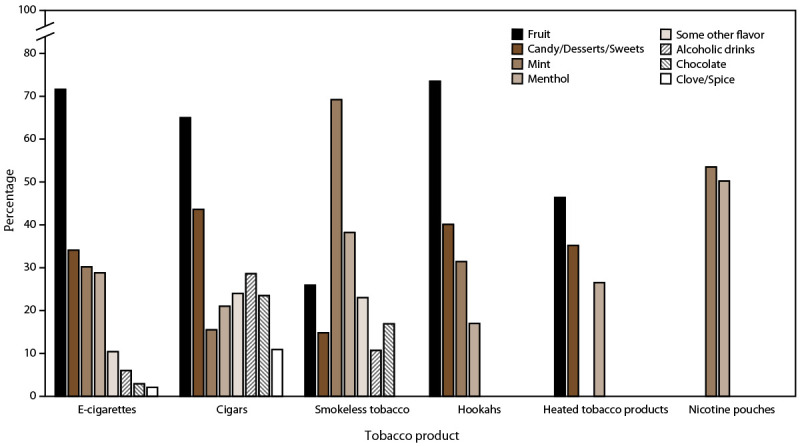
Flavor types* used among middle and high school students who reported current (past 30-day) use of flavored tobacco products, by product^†,§,¶^ — National Youth Tobacco Survey, United States, 2021 **Abbreviation:** e-cigarettes = electronic cigarettes. * For each respective tobacco product excluding cigarettes (e-cigarettes, cigars [cigars, cigarillos, or little cigars], smokeless tobacco [chewing tobacco, snuff, or dip; snus; dissolvable tobacco products], hookahs, pipe tobacco, heated tobacco products, nicotine pouches), current (past 30-day) users were asked, “Were any of the [tobacco product] that you used in the past 30 days flavored to taste like menthol, mint, clove or spice, alcohol (wine, cognac), candy, fruit, chocolate, or any other flavor?” (response options were “yes,” “no,” or “don’t know”). Those who responded yes were then asked, “What flavors were the [tobacco product] that you have used in the past 30 days? (Select one or more).” Response options were menthol, mint, clove or spice, alcoholic drinks (such as wine, cognac, margarita, or other cocktails), candy, desserts, or other sweets, fruit, chocolate, and some other flavor not listed here (write-in responses). Write-in responses were examined and recoded to a prespecified response option where applicable. ^†^ For cigarettes, flavored use refers to menthol cigarette use only. All other flavor types for cigarettes are not applicable. Menthol cigarette use is omitted from this figure. ^§^ For smokeless tobacco, flavor types were assessed separately among flavored chewing tobacco, snuff, or dip; snus; and dissolvable tobacco product users. Flavor types were then assessed as a composite among current users of chewing, snuff, dip, snus or dissolvable tobacco products. ^¶^ Data are not shown as they were statistically unreliable because of unweighted denominator <50 or a relative standard error >30%: smokeless tobacco: clove or spice; hookahs: some other flavor, alcoholic drinks, chocolate, clove or spice; heated tobacco products: mint, some other flavor, alcoholic drinks, chocolate, clove or spice; nicotine pouches: fruit; candy, desserts, or sweets; some other flavor; alcoholic drinks; chocolate; or clove or spice. All estimates of flavor types used for pipe tobacco were statistically unreliable and are omitted from this figure.

Among students who currently used each flavored tobacco product, fruit was the most commonly reported flavor type for e-cigarettes (71.6%), hookahs (73.5%), cigars (65.0%), and HTPs (46.4%) (Figure 2) (Supplementary Table 1; https://stacks.cdc.gov/view/cdc/114659); mint was the most commonly reported flavor type for smokeless tobacco (69.2%) and nicotine pouches (53.5%). Menthol use was reported as 50.2% for nicotine pouches, 38.8% for cigarettes, 38.2% for smokeless tobacco, 28.8% for e-cigarettes, 26.5% for HTPs, 21.0% for cigars, and 17.0% for hookahs.

### E-Cigarette Device Type

**FIGURE 3:**
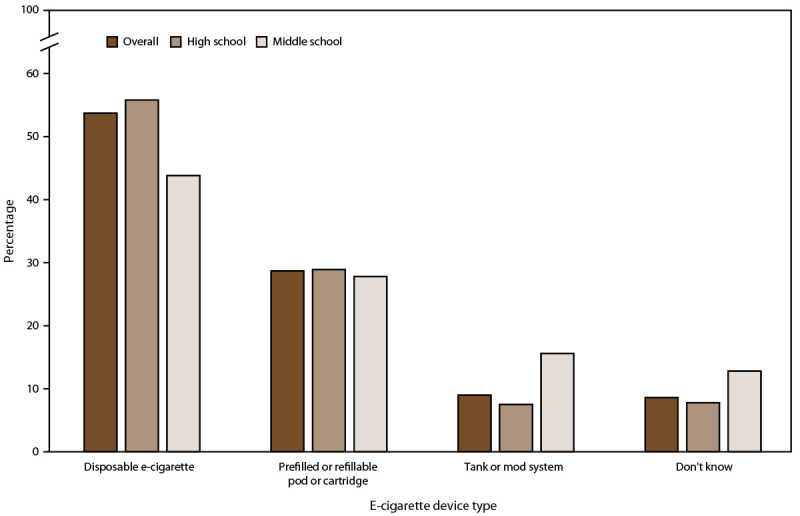
E-cigarette device types* reported among middle and high school students who reported current (past 30-day) use of e-cigarettes, overall and by school level — National Youth Tobacco Survey, United States, 2021 **Abbreviation:** e-cigarettes = electronic cigarettes. * Device type among current e-cigarette users was assessed by the question, “Which of the following best describes the type of e-cigarette you have used in the past 30 days? If you have used more than one type, please think about the one you use most often. Response options included the following: “a disposable e-cigarette (for example, Puff Bar or Stig),” “an e-cigarette that uses pre-filled or refillable pods or cartridges (for example, Juul, Smok, or Suorin),” “an e-cigarette with a tank that you refill with liquids (including mod systems that can be customized by the user),” and “I don’t know the type.” ^†^ Past 30-day use of e-cigarettes was determined by asking, “During the past 30 days, on how many days did you use e-cigarettes?” Those indicating use on ≥1 day of the past 30 days were considered current user of the respective product.

Among students who currently used e-cigarettes, disposable e-cigarettes were the most commonly used device type (overall: 53.7%, 1.08 million; high school: 55.8%, 940,000; middle school: 43.8%, 130,000), followed by prefilled or refillable pods or cartridges (overall: 28.7%, 570,000; high school: 28.9%, 480,000; middle school: 27.8%, 80,000), and tanks or mod systems (overall: 9.0%, 180,000; high school: 7.5%, 120,000; middle school: 15.6%, 40,000) (Figure 3). Estimates of e-cigarette device type by sex and race or ethnicity are provided (Supplementary Table 2; https://stacks.cdc.gov/view/cdc/114659).

### Reasons for E-Cigarette Use

Among students who ever used e-cigarettes, the most common reasons for first use were “a friend used them” (57.8%), “I was curious about them” (47.6%), “I was feeling anxious, stressed, or depressed” (25.1%), and “to get a high or buzz from nicotine” (23.3%) (Table 6). The most commonly reported reason for first use among high school students was “a friend used them” (60.6%) and was “I was curious about them” (49.4%) for middle school students.

**TABLE 6:** Reasons for first e-cigarette use* among middle and high school students who reported ever using e-cigarettes, and reasons for current (past 30-day) e-cigarette use^†^ among middle and high school students who reported currently using e-cigarettes, overall and by school level — National Youth Tobacco Survey, United States, 2021

Characteristic	Among ever e-cigarette users		Among current e-cigarette users	
	% (95% CI)	Estimated no. of users^§^	% (95% CI)	Estimated no. of users^§^
**Overall**				
A friend [used/uses] them	57.8 (55.0–60.4)	2,990,000	28.3 (25.4–31.5)	560,000
I [was/am] curious about them	47.6 (45.4–49.9)	2,460,000	10.3 (8.5–12.6)	200,000
I [was/am] feeling anxious, stressed, or depressed	25.1 (23.0–27.4)	1,300,000	43.4 (39.4–47.4)	860,000
To get a high or buzz from nicotine	23.3 (21.5–25.2)	1,200,000	42.8 (38.4–47.4)	850,000
A family member [used/uses] them	18.6 (16.5–20.9)	960,000	8.7 (7.0–10.8)	170,000
I [could/can] use them to do tricks	16.5 (14.5–18.7)	850,000	20.0 (17.5–22.7)	390,000
They [were/are] available in flavor, such as menthol, mint, candy, fruit, or chocolate	13.5 (12.0–15.0)	690,000	13.2 (11.1–15.6)	260,000
I [could/can] use them unnoticed at home or at school	10.8 (9.5–12.4)	560,000	13.0 (10.9–15.3)	250,000
They are less harmful than other forms of tobacco, such as cigarettes	8.3 (7.1–9.6)	420,000	10.3 (8.3–12.7)	200,000
They [were/are] easier to get that other tobacco products, such as cigarettes	4.8 (3.8–6.0)	240,000	6.0 (4.7–7.7)	120,000
I’ve seen people on TV, online, or in movies use them	4.5 (3.7–5.6)	230,000	2.9 (2.1–4.0)	50,000
To try to quit using other tobacco products, such as cigarettes	2.5 (1.9–3.4)	130,000	4.6 (3.5–6.1)	90,000
They cost less than other tobacco products, such as cigarettes	2.2 (1.6–2.9)	110,000	4.7 (3.4–6.6)	90,000
Some other reason	10.6 (9.2–12.1)	540,000	19.5 (16.9–22.5)	380,000
**High school (grades 9–12)**				
A friend [used/uses] them	60.6 (57.7–63.4)	2,630,000	27.6 (24.6–30.8)	460,000
I [was/am] curious about them	47.2 (44.7–49.7)	2,050,000	9.0 (7.0–11.5)	150,000
I [was/am] feeling anxious, stressed, or depressed	24.5 (22.0–27.2)	1,060,000	43.2 (38.5–48.1)	720,000
To get a high or buzz from nicotine	24.9 (22.9–26.9)	1,080,000	45.3 (40.4–50.3)	750,000
A family member [used/uses] them	16.0 (13.9–18.4)	690,000	6.3 (4.8–8.3)	100,000
I [could/can] use them to do tricks	16.4 (14.2–18.8)	710,000	19.5 (16.9–22.5)	320,000
They [were/are] available in flavor, such as menthol, mint, candy, fruit, or chocolate	13.2 (11.7–14.8)	570,000	13.2 (11.1–15.7)	220,000
I [could/can] use them unnoticed at home or at school	11.2 (9.6–13.0)	480,000	13.9 (11.7–16.5)	230,000
They are less harmful than other forms of tobacco, such as cigarettes	8.3 (7.2–9.6)	360,000	10.3 (8.2–13.0)	170,000
They [were/are] easier to get that other tobacco products, such as cigarettes	4.5 (3.4–6.0)	190,000	5.5 (4.1–7.3)	90,000
I’ve seen people on TV, online, or in movies use them	4.4 (3.5–5.6)	190,000	2.5 (1.7–3.6)	40,000
To try to quit using other tobacco products, such as cigarettes	2.3 (1.6–3.3)	100,000	4.1 (2.9–5.9)	60,000
They cost less than other tobacco products, such as cigarettes	1.9 (1.3–2.7)	80,000	4.1 (2.8–6.1)	60,000
Some other reason	9.0 (7.6–10.7)	390,000	19.0 (16.1–22.4)	310,000
**Middle school (grades 6–8)**				
A friend [used/uses] them	42.6 (37.8–47.7)	340,000	31.7 (24.1–40.4)	90,000
I [was/am] curious about them	49.4 (44.1–54.9)	400,000	17.8 (13.4–23.3)	50,000
I [was/am] feeling anxious, stressed, or depressed	28.1 (24.6–31.9)	230,000	45.0 (38.8–51.4)	140,000
To get a high or buzz from nicotine	14.5 (11.0–19.0)	110,000	29.2 (22.7–36.6)	90,000
A family member [used/uses] them	32.0 (28.2–36.1)	260,000	21.0 (15.9–27.2)	60,000
I [could/can] use them to do tricks	16.3 (12.9–20.4)	130,000	22.2 (15.3–31.1)	60,000
They [were/are] available in flavor, such as menthol, mint, candy, fruit, or chocolate	14.2 (10.8–18.5)	110,000	12.1 (7.8–18.1)	30,000
I [could/can] use them unnoticed at home or at school	8.3 (6.0–11.4)	60,000	8.1 (4.7–13.7)	20,000
They are less harmful than other forms of tobacco, such as cigarettes	7.7 (5.4–10.9)	60,000	10.0 (6.3–15.7)	30,000
They [were/are] easier to get that other tobacco products, such as cigarettes	6.0 (3.9–9.0)	40,000	7.8 (4.7–12.6)	20,000
I’ve seen people on TV, online, or in movies use them	4.9 (3.5–6.7)	30,000	—^¶^	—
To try to quit using other tobacco products, such	—	—	6.0 (3.4–10.5)	10,000
They cost less than other tobacco products, such as cigarettes	—	—	7.5 (4.3–12.9)	20,000
Some other reason	18.4 (15.1–22.3)	150,000	22.0 (14.9–31.1)	60,000

**Abbreviations: **e-cigarettes = electronic cigarettes; TV = television.

* Responses to the question, “Why did you first use an e-cigarette? (Select one or more)” were assessed among students who reported “yes” to the question, “Have you ever used an e-cigarette, even once or twice?” Respondents indicating “some other reason” could specify through write-in option; write-ins were not examined in this report (n = 275).

^† ^Responses to the question, “Why did you currently use e-cigarettes? (Select one or more)” were assessed among students who reported using e-cigarettes on 1–30 days, from the question, “During the past 30 days, on how many days did you use e-cigarettes? Respondents indicating “some other reason” could specify through write-in option; write-ins were not examined in this report (n = 195).

^§ ^Estimated weighted total numbers of ever and current e-cigarette users were rounded down to the nearest 10,000 persons. Overall estimates might not directly total to sums of corresponding subgroup estimates because of rounding or inclusion of students who did not self-report grade level.

^¶^ Data were statistically unreliable because of unweighted denominator <50 or a relative standard error >30%.

Among students who currently used e-cigarettes, the most common reasons for current use were “I am feeling anxious, stressed, or depressed” (43.4%), “to get a high or buzz from nicotine” (42.8%), “a friend uses them” (28.3%), and “I can use them to do tricks” (20.0%) (Table 6). The most commonly reported reason for current use was “to get a high or buzz from nicotine” (45.3%) among high school students and “I am feeling anxious, stressed, or depressed” (45.0%) among middle school students.

### Access to Tobacco Products

Among students who currently used any tobacco product, during the past 30 days, 32.8% got their products from a friend, 31.3% bought the products themselves, 28.8% had someone else buy the products for them, and 26.3% had someone offer the products to them (Table 7).

**TABLE 7:** Access to tobacco products*^,†^ among middle and high school students who reported current (past 30-day) use of each tobacco product — National Youth Tobacco Survey, United States, 2021

Access to each tobacco product	Tobacco product								
	Any tobacco^§^	E-cigarettes	Cigarettes	Cigars	Smokeless tobacco^¶^	Hookahs	Pipe tobacco	Heated tobacco products	Nicotine pouch
	% (95% CI)	% (95% CI)	% (95% CI)	% (95% CI)	% (95% CI)	% (95% CI)	% (95% CI)	% (95% CI)	% (95% CI)
**How did you get your [tobacco product]?**									
I got them from a friend	32.8 (29.7–36.0)	32.3 (28.7–36.2)	18.4 (13.5–24.7)	22.1 (16.2–29.2)	26.3 (18.5–35.9)	23.9 (16.8–33.0)	—**	23.4 (12.8–39.0)	22.7 (14.9–33.0)
I bought them myself	31.3 (28.5–34.2)	31.1 (27.4–35.0)	22.0 (16.3–29.0)	29.8 (23.4–37.0)	24.1 (17.6–32.1)	18.3 (12.8–25.5)	—	14.6 (8.8–23.2)	—
I had someone else buy them for me	28.8 (26.0–31.8)	28.7 (25.7–31.8)	13.6 (9.4–19.1)	23.7 (17.7–31.1)	26.8 (19.8–35.3)	15.7 (9.9–24.2)	—	24.5 (14.8–37.7)	17.7 (11.2–27.0)
Someone offered them to me	26.3 (23.1–29.7)	21.7 (19.2–24.4)	16.4 (10.7–24.2)	22.8 (17.3–29.4)	27.8 (19.5–37.9)	26.5 (18.8–35.8)	32.0 (19.5–47.8)	23.7 (15.6–34.2)	24.6 (17.2–33.9)
I asked someone to give me some	19.4 (16.6–22.6)	16.3 (13.8–19.2)	20.2 (14.6–27.3)	14.3 (8.9–22.2)	23.6 (15.4–34.4)	22.1 (13.0–35.0)	22.8 (12.2–38.6)	—	13.3 (7.1–23.3)
I got them from a family member	14.5 (12.3–17.1)	10.2 (8.2–12.6)	17.5 (12.7–23.6)	16.7 (12.1–22.6)	20.5 (13.7–29.5)	19.5 (11.9–30.3)	—	15.8 (8.5–27.7)	—
I took them from a store or another person	6.0 (4.6–7.7)	3.3 (2.4–4.6)	12.2 (8.0–18.2)	6.5 (3.8–10.7)	9.2 (5.2–15.7)	—	—	—	—
I got them in some other way	21.3 (19.0–23.8)	15.7 (13.1–18.6)	21.8 (16.3–28.5)	22.7 (16.6–30.3)	23.5 (16.7–31.9)	24.6 (17.5–33.3)	23.4 (15.3–34.1)	27.5 (20.1–36.5)	20.6 (11.7–33.8)
**Where did you buy your [tobacco product]?**									
I did not buy them	48.6 (45.9–51.4)	37.2 (34.2–40.3)	48.0 (40.9–55.2)	33.2 (26.1–41.1)	33.4 (24.2–44.0)	54.2 (45.7–62.5)	37.0 (25.0–50.8)	33.2 (24.2–43.5)	34.3 (24.3–46.0)
Bought them from another person (friend, family member, someone else)	22.5 (19.4–26.1)	21.5 (18.2–25.2)	14.7 (10.3–20.5)	13.4 (9.2–19.0)	21.0 (14.4–29.4)	—	23.6 (13.2–38.6)	24.2 (15.5–35.8)	13.9 (7.9–23.3)
A vape shop or tobacco shop	20.2 (16.8–24.0)	22.2 (18.3–26.6)	7.0 (4.1–11.7)	12.2 (8.2–17.8)	—	—	—	—	—
A gas station, convenience store	19.6 (16.9–22.7)	17.7 (14.4–21.5)	17.1 (12.1–23.6)	34.1 (26.2–42.9)	17.9 (11.2–27.3)	8.5 (4.7–15.0)	—	—	—
A drugstore	4.8 (3.5–6.5)	3.7 (2.6–5.4)	—	—	5.0 (2.8–8.8)	—	—	—	—
A grocery store	4.8 (3.6–6.4)	2.6 (1.6–4.0)	5.3 (3.0–9.1)	—	10.3 (5.8–17.7)	—	—	—	—
Through the mail	3.6 (2.4–5.4)	2.2 (1.3–3.8)	—	—	6.7 (3.7–11.9)	—	—	—	—
On the Internet	3.5 (2.5–4.8)	2.9 (1.9–4.5)	—	—	—	—	—	—	—
A mall or shopping center kiosk or stand	3.2 (2.1–4.9)	1.7 (1.0–2.8)	—	—	—	—	—	—	—
A vending machine	2.9 (2.1–4.2)	1.8 (1.2–2.8)	—	—	—	6.9 (3.7–12.2)	—	—	—
Through a delivery service	2.4 (1.6–3.5)	1.6 (0.9–2.5)	—	—	—	—	—	—	—
Some other place	15.8 (13.8–18.1)	12.3 (10.5–14.4)	17.9 (12.6–24.8)	14.5 (9.6–21.3)	22.2 (16.3–29.3)	18.9 (11.0–30.4)	—	19.8 (13.2–28.7)	21.5 (13.0–33.4)

**Abbreviation: **e-cigarettes = electronic cigarettes.

* “During the past 30 days, how did you get your [e-cigarette devices, pods, cartridges, or e-liquid refills; cigarettes; cigars, cigarillos, or little cigars; chewing tobacco, snuff, or dip; hookahs; pipe tobacco; snus; dissolvable tobacco products; heated tobacco products; nicotine pouches]? (Select one or more).”

^†^ “During the past 30 days, where did you buy your [e-cigarette devices, pods, cartridges, or e-liquid refills; cigarettes; cigars, cigarillos, or little cigars; chewing tobacco, snuff, or dip; hookahs; pipe tobacco; snus; dissolvable tobacco products; heated tobacco products; nicotine pouches]

^§^ Any tobacco use includes use of e-cigarettes, cigarettes, cigars (cigars, cigarillos, or little cigars), smokeless tobacco (chewing tobacco, snuff, or dip; snus; or dissolvable tobacco products), hookahs, pipe tobacco, bidis (small brown cigarettes wrapped in a leaf), heated tobacco products and nicotine pouches on ≥1 day during the past 30 days.

^¶^ Access (“how did you get …” and “where did you buy …”) during the past 30 days was assessed separately for smokeless tobacco products (chewing tobacco, snuff, or dip; snus; or dissolvable tobacco products). Access was then recoded as a smokeless composite among students who reported past 30-day use of one or more smokeless tobacco product.

** Data were statistically unreliable because of unweighted denominator <50 or a relative standard error >30%.

By purchase locations, 22.5% of students who currently used tobacco products reported purchasing from another person (friend, family member, or someone else), 20.2% from vape shops or tobacco shops, and 19.6% from gas stations or convenience stores; 48.6% of current tobacco product users did not purchase the products they used in the past 30 days.

### Recognition of Public Education Campaigns Against Tobacco Product Use

Among all students, 75.2% (high school: 79.4%; middle school: 69.8%) had seen or heard at least one public education campaign against tobacco product use within the past year (Table 8). Recognition was 78.3% among males, 71.9% among females, and ranged from 67.6% among students who were non-Hispanic other race to 79.2% among students who were non-Hispanic White. Recognition was 60.9% for “The Real Cost,” 39.7% for “Truth,” 19.8% for “Tips” or “Tips from Former Smokers,” 6.9% for “Fresh Empire,” 4.8% for “This Free Life,” and 11.7% for “some other ad.”

**TABLE 8:** Recognition of public education campaign advertisements against tobacco product use in the past 12 months* among middle and high school students, overall and by school level — National Youth Tobacco Survey, United States, 2021

Characteristic	Sex		Race and ethnicity				Total	
	Male	Female	White, non-Hispanic	Black, non-Hispanic	Hispanic^†^	Other, non-Hispanic		
	% (95% CI)	% (95% CI)	% (95% CI)	% (95% CI)	% (95% CI)	% (95% CI)	% (95% CI)	Estimated no.^§^
**Overall**								
One or more	78.3 (76.5–79.9)	71.9 (70.3–73.5)	79.2 (77.6–80.8)	75.8 (73.2–78.3)	69.8 (67.7–71.7)	67.6 (62.4–72.3)	**75.2 (73.7–76.6)**	**19,560,000**
The Real Cost	65.0 (62.7–67.3)	56.5 (54.4–58.5)	66.5 (64.5–68.6)	54.9 (51.3–58.4)	55.0 (52.9–57.1)	54.8 (49.0–60.5)	**60.9 (58.9–62.8)**	**15,830,000**
Truth	41.7 (39.4–43.9)	37.7 (35.8–39.6)	43.9 (41.5–46.3)	38.2 (34.7–41.8)	35.9 (34.2–37.6)	29.9 (26.6–33.5)	**39.7 (37.9–41.6)**	**10,200,000**
Tips, Tips from Former Smokers	19.9 (18.6–21.2)	19.7 (18.5–21.1)	21.8 (20.4–23.2)	20.8 (18.3–23.5)	16.9 (15.7–18.2)	16.5 (12.9–20.9)	**19.8 (18.7–20.9)**	**5,080,000**
Fresh Empire	7.5 (5.5–10.1)	6.3 (5.5–7.3)	5.4 (4.1–7.0)	17.6 (13.9–21.9)	5.9 (5.1–6.9)	4.7 (3.1–7.0)	**6.9 (5.7–8.4)**	**1,780,000**
This Free Life	5.0 (4.4–5.6)	4.5 (3.9–5.3)	4.7 (3.9–5.5)	5.8 (4.6–7.2)	4.7 (3.9–5.6)	4.7 (3.2–6.8)	**4.8 (4.3–5.3)**	**1,220,000**
Some other advertisement	12.0 (11.0–13.0)	11.3 (10.4–12.3)	11.0 (10.0–12.0)	13.7 (11.9–15.6)	12.2 (11.2–13.3)	10.6 (8.5–13.1)	**11.7 (11.0–12.4)**	**2,990,000**
**High school (grades 9–12)**								
One or more	82.4 (80.4–84.3)	76.1 (73.9–78.2)	84.1 (82.5–85.5)	78.0 (74.6–81.1)	71.3 (68.4–74.1)	74.0 (68.2–79.0)	**79.4 (77.7–81.1)**	**11,570,000**
The Real Cost	69.4 (66.6–72.1)	61.7 (59.2–64.2)	71.9 (69.5–74.2)	56.6 (52.4–60.6)	57.9 (55.4–60.3)	61.3 (52.6–69.3)	**65.8 (63.4–68.0)**	**9,560,000**
Truth	48.4 (45.7–51.0)	44.2 (41.8–46.7)	51.4 (49.1–53.7)	40.8 (36.3–45.5)	40.3 (37.5–43.1)	37.0 (30.7–43.8)	**46.4 (44.1–48.7)**	**6,680,000**
Tips, Tips from Former Smokers	21.7 (20.0–23.5)	20.2 (18.3–22.2)	23.2 (21.5–25.0)	21.6 (18.1–25.5)	17.5 (15.7–19.4)	14.3 (10.2–19.7)	**21.0 (19.6–22.5)**	**3,020,000**
Fresh Empire	10.1 (7.0–14.3)	7.5 (6.2–8.9)	6.7 (4.9–9.2)	22.8 (17.3–29.4)	7.1 (5.7–8.8)	6.2 (3.9–9.6)	**8.8 (6.9–11.2)**	**1,270,000**
This Free Life	5.2 (4.4–6.0)	4.7 (3.9–5.7)	4.6 (3.8–5.6)	5.0 (3.8–6.6)	5.4 (4.4–6.6)	6.1 (4.1–9.0)	**4.9 (4.4–5.6)**	**710,000**
Some other advertisement	10.7 (9.6–12.0)	8.6 (7.6–9.8)	9.0 (7.9–10.3)	12.3 (9.9–15.2)	10.0 (8.7–11.6)	10.1 (6.9–14.5)	**9.7 (8.9–10.6)**	**1,400,000**
**Middle school (grades 6–8)**								
One or more	72.9 (70.6–75.0)	66.8 (64.7–68.8)	72.4 (70.1–74.6)	73.1 (69.2–76.7)	68.0 (65.1–70.6)	62.2 (55.8–68.2)	**69.8 (68.0–71.6)**	**7,940,000**
The Real Cost	59.5 (56.5–62.4)	50.1 (47.4–52.8)	59.0 (56.5–61.5)	52.8 (46.4–59.1)	51.8 (47.7–55.8)	49.4 (43.5–55.3)	**54.8 (52.4–57.3)**	**6,250,000**
Truth	32.9 (30.9–34.9)	29.6 (27.8–31.5)	33.0 (30.8–35.2)	34.6 (30.5–38.8)	30.8 (28.3–33.3)	23.8 (20.2–27.8)	**31.2 (29.7–32.8)**	**3,490,000**
Tips, Tips from Former Smokers	17.5 (16.0–19.1)	19.2 (17.9–20.6)	19.7 (18.0–21.5)	19.8 (16.8–23.1)	16.3 (14.6–18.0)	18.5 (14.7–23.0)	**18.3 (17.1–19.5)**	**2,040,000**
Fresh Empire	4.1 (3.4–5.0)	5.0 (4.0–6.1)	3.4 (2.6–4.6)	10.5 (7.8–14.1)	4.6 (3.4–6.0)	—^¶^	**4.5 (3.8–5.3)**	**500,000**
This Free Life	4.7 (3.8–5.8)	4.4 (3.4–5.5)	4.7 (3.5–6.3)	6.8 (4.9–9.5)	3.9 (3.0–4.9)	—	**4.5 (3.8–5.4)**	**500,000**
Some other advertisement	13.6 (12.1–15.1)	14.6 (13.4–16.0)	13.7 (12.3–15.2)	15.6 (13.4–18.1)	14.6 (13.0–16.4)	11.0 (9.1–13.3)	**14.1 (13.2–15.0)**	**1,580,000**

**Abbreviation: **e-cigarettes = electronic cigarettes.

* All respondents were asked, “In the past 12 months, have you seen or heard “The Real Cost,” on television, the internet, social media, or radio as part of ads about tobacco?” Response options included “yes,” “no,” and “not sure.” Those who selected “yes” were considered to have recognized the advertisement. Additionally, all respondents were asked, “In the past 12 months, have you seen or heard any other ads against tobacco with the following names or slogans on television, the internet, social media, or on the radio? (Select one or more).” Response options included : “Truth,” “Tips or Tips from Former Smokers,” “Fresh Empire,” “This Free Life,” “some other ad”; or exclusively select “I haven’t seen or heard any of these ads.” A composite of having recognized at least one ad was also assessed. Those who indicated “some other ad” could specify with a write-in response; analysis of these write-in responses (n = 892) was not included in this report.

^†^ Hispanic persons could be of any race.

^§ ^Estimated weighted total number of flavored tobacco product users was rounded down to the nearest 10,000 persons. Overall estimates might not directly total to sums of corresponding subgroup estimates because of rounding or inclusion of students who did not self-report grade level.

^¶^ Data were statistically unreliable because of unweighted denominator <50 or a relative standard error >30%.

### Exposure to Tobacco Product Marketing

Among students who reported contact with a potential source of tobacco product marketing, 75.7% (high school: 79.6%; middle school: 70.7%) reported exposure to any tobacco product marketing from one or more of these sources (Table 9). The prevalence of exposure was 65.4% among students who reported going to retail stores, 43.9% among those who reported using the Internet, 34.0% among those who reported reading newspapers or magazines, and 30.3% among those who reported watching television or streaming services or going to the movies. Overall, 70.3% of middle and high school students reported exposure to e-cigarette marketing specifically, whereas 59.1% reported exposure to cigarette or other tobacco product marketing.

**TABLE 9:** Exposure* to sources of tobacco product marketing (advertisements or promotions) among middle and high school students who reported contact with each source, overall and by school level, sex, and race and ethnicity — National Youth Tobacco Survey, United States, 2021

Characteristic	Retail stores^†^	Internet^§^	Television, streaming services, or movies^¶^	Newspapers or magazines**	Any source^††^
**% (95% CI)**	**% (95% CI)**	**% (95% CI)**	**% (95% CI)**	**% (95% CI)**
**Prevalence of exposure to any tobacco product marketing (e-cigarettes, cigarettes, and other tobacco products)**					
**Overall**	**65.4 (63.6–67.1)**	**43.9 (42.3–45.4)**	**30.3 (28.9–31.8)**	**34.0 (32.6–35.4)**	**75.7 (74.3–77.0)**
**Estimated no.^§§^**	**16,240,000**	**11,050,000**	**7,460,000**	**4,830,000**	**19,210,000**
**Sex**					
Male	64.2 (62.3–66.1)	40.9 (39.2–42.6)	27.4 (25.9–29.0)	33.4 (31.5–35.3)	**73.2 (71.7–74.8)**
Female	66.6 (64.6–68.6)	46.9 (45.3–48.6)	33.3 (31.6–34.9)	34.6 (32.8–36.4)	**78.3 (76.8–79.8)**
**Race and ethnicity**					
White, non-Hispanic	69.9 (67.7–71.9)	42.8 (40.6–45.0)	27.8 (26.0–29.8)	33.1 (31.1–35.0)	**78.1 (76.5–79.6)**
Black, non-Hispanic	61.7 (59.2–64.1)	49.2 (47.0–51.3)	39.7 (36.5–43.1)	36.2 (33.4–39.2)	**76.0 (73.7–78.3)**
Hispanic^¶¶^	61.3 (59.6–63.0)	45.0 (42.9–47.0)	31.9 (30.0–33.8)	35.0 (32.7–37.3)	**73.9 (72.3–75.3)**
Other, non-Hispanic	53.9 (50.4–57.5)	40.6 (36.0–45.3)	27.7 (24.5–31.1)	34.1 (28.9–39.7)	**66.0 (62.3–69.5)**
**School level**					
High school (grades 9–12)	69.7 (67.4–71.9)	45.8 (43.8–47.8)	32.6 (30.4–34.9)	35.6 (33.7–37.5)	**79.6 (78.0–81.1)**
Middle school (grades 6–8)	59.8 (57.7–61.9)	41.2 (39.3–43.2)	27.3 (25.5–29.2)	31.6 (29.5–33.8)	**70.7 (69.1–72.3)**
Exposure to e-cigarette marketing***					
**Overall**	**58.7 (56.9–60.5)**	**36.0 (34.8–37.2)**	**21.7 (20.5–23.0)**	**28.7 (27.2–30.1)**	**70.3 (68.8–71.7)**
**Estimated no.**	**14,370,000**	**8,970,000**	**5,240,000**	**3,500,000**	**17,770,000**
**Sex**					
Male	57.5 (55.6–59.4)	33.7 (32.3–35.1)	19.8 (18.5–21.2)	28.3 (26.3–30.4)	**67.7 (66.1–69.3)**
Female	60.0 (57.9–62.0)	38.4 (37.0–39.8)	23.6 (22.2–25.0)	29.1 (27.2–31.0)	**73.0 (71.3–74.6)**
**Race and ethnicity**					
White, non-Hispanic	63.6 (61.5–65.7)	35.9 (34.2–37.6)	19.3 (17.7–21.1)	27.7 (25.8–29.7)	**73.1 (71.4–74.7)**
Black, non-Hispanic	52.4 (49.8–55.0)	38.6 (36.7–40.5)	30.4 (27.7–33.2)	30.6 (27.2–34.3)	**70.4 (68.0–72.7)**
Hispanic	54.7 (52.9–56.6)	36.2 (34.7–37.8)	23.1 (21.6–24.7)	30.4 (28.0–32.9)	**67.7 (65.8–69.4)**
Other, non-Hispanic	47.3 (43.5–51.1)	33.1 (29.0–37.5)	20.2 (17.2–23.5)	26.6 (21.4–32.5)	**60.0 (56.1–63.8)**
**School level**					
High school (grades 9–12)	63.2 (60.8–65.6)	38.1 (36.4–39.8)	23.3 (21.5–25.3)	29.4 (27.4–31.6)	**74.3 (72.6–75.9)**
Middle school (grades 6–8)	52.8 (50.9–54.8)	33.2 (31.6–34.8)	19.5 (18.1–21.0)	27.4 (25.4–29.6)	**65.1 (63.4–66.7)**
**Exposure to cigarette and other tobacco product marketing^†††^**					
**Overall**	**50.9 (49.1–52.7)**	**27.0 (25.5–28.5)**	**21.4 (20.1–22.7)**	**25.5 (24.3–26.8)**	**59.1 (57.2–60.9)**
**Estimated no.**	**12,100,000**	**6,500,000**	**5,040,000**	**2,920,000**	**14,550,000**
**Sex**					
Male	49.6 (47.8–51.4)	25.1 (23.4–26.8)	19.4 (18.0–20.8)	24.7 (23.2–26.1)	**56.8 (54.9–58.7)**
Female	52.4 (50.2–54.6)	28.9 (27.0–30.8)	23.4 (21.9–25.0)	26.3 (24.6–28.2)	**61.5 (59.1–63.9)**
**Race and ethnicity**					
White, non-Hispanic	54.9 (52.5–57.2)	25.6 (23.6–27.6)	19.3 (17.8–20.9)	25.2 (23.5–26.9)	**61.8 (59.3–64.2)**
Black, non-Hispanic	47.4 (44.6–50.1)	32.5 (30.3–34.7)	28.0 (25.4–30.8)	28.4 (26.1–30.9)	**58.9 (56.3–61.4)**
Hispanic	48.0 (46.2–49.8)	28.0 (26.1–29.9)	23.3 (21.6–25.0)	24.9 (22.7–27.4)	**56.9 (55.1–58.7)**
Other, non-Hispanic	40.2 (36.7–43.7)	25.4 (21.5–29.7)	18.8 (15.7–22.4)	24.9 (20.8–29.5)	**49.3 (44.9–53.6)**
**School level**					
High school (grades 9–12)	53.9 (51.6–56.2)	27.3 (25.3–29.3)	23.3 (21.4–25.2)	27.0 (25.3–28.9)	**61.7 (59.4–63.9)**
Middle school (grades 6–8)	47.1 (44.8–49.4)	26.4 (24.7–28.2)	19.0 (17.5–20.5)	23.2 (21.1–25.4)	**55.7 (53.4–57.9)**

**Abbreviations: **e-cigarettes = electronic cigarettes; TV = television.

* Exposure to tobacco product marketing was assessed for each of four marketing sources (retail stores; Internet; television, streaming sources, or movies; and newspapers or magazines) and any source combined. For each source, exposure was assessed separately for 1) e-cigarettes and 2) cigarettes or other tobacco products. A composite measure of exposure to any tobacco product marketing was also assessed. Prevalence of exposure to tobacco product marketing was restricted to students who reported using each source.

^†^ Assessed by the questions, “When you go to a convenience store, supermarket, or gas station, how often do you see ads or promotions for (e-cigarettes; cigarettes or other tobacco products)?” Response options for both questions included, “I never go to a convenience store, supermarket, or gas station,” “never,” “rarely,” “sometimes,” “most of the time,” and “always.” Respondents were categorized as exposed if they reported “sometimes,” “most of the time,” or “always”; respondents were categorized as unexposed if they reported “never” or “rarely.” Persons who reported “I never go to a convenience store, supermarket, or gas station” were set to missing and excluded from product-specific analyses. Total unweighted denominators included n = 18,065 (e-cigarettes) and n = 17,571 (cigarettes or other tobacco products). A composite measure of exposure to any tobacco product marketing when going to retail stores (e-cigarettes and cigarettes or other tobacco products) was assessed among respondents who had visited a retail outlet and had nonmissing data for at least one measure (n = 18,361).

^§^ Assessed by the questions, “When you are using the Internet, how often do you see ads or promotions for (e-cigarettes; cigarettes or other tobacco products)?” Response options for both questions included, “I do not use the Internet,” “never,” “rarely,” “sometimes,” “most of the time,” and “always.” Respondents were categorized as exposed if they reported “sometimes,” “most of the time,” or “always”; respondents were categorized as unexposed if they reported “never” or “rarely.” Persons who reported “I do not use the Internet” were set to missing and excluded from product-specific analyses. Total unweighted denominators included n = 18,408 (e-cigarettes) and n = 17,858 (cigarettes or other tobacco products). A composite measure of exposure to any tobacco product marketing when using the Internet (e-cigarettes and cigarettes or other tobacco products) was assessed among respondents who had used the Internet and had nonmissing data for at least one measure (n = 18,627).

^¶^ Assessed by the questions, “When you watch TV or streaming services (such as Netflix, Hulu, or Amazon Prime), or go to the movies, how often do you see ads or promotions for (e-cigarettes; cigarettes or other tobacco products)?” Response options for both questions included, “I do not watch TV or streaming services, or go to the movies,” “never,” “rarely,” “sometimes,” “most of the time,” and “always.” Respondents were categorized as exposed if they reported “sometimes,” “most of the time,” or “always”; respondents were categorized as unexposed if they reported “never” or “rarely.” Persons who reported “I do not watch TV or streaming services or go to the movies” were set to missing and excluded from product-specific analyses. Total unweighted denominators for exposure to tobacco product marketing included n = 17,883 (e-cigarettes) and n = 17,428 (cigarettes or other tobacco products). A composite measure of exposure to any tobacco product marketing when watching television or streaming services or going to the movies (e-cigarettes and cigarettes or other tobacco products) was assessed among respondents who had watched TV or streaming services or who went to the movies and had nonmissing data for at least one measure (n = 18,215).

** Assessed by the questions, “When you read newspapers or magazines, how often do you see ads or promotions for (e-cigarettes; cigarettes or other tobacco products)?” Response options for both questions included, “I do not read newspapers or magazines,” “never,” “rarely,” “sometimes,” “most of the time,” and “always.” Respondents were categorized as exposed if they reported “sometimes,” “most of the time,” or “always”; respondents were categorized as unexposed if they reported “never” or “rarely.” Persons who reported “I do not read newspapers or magazines” were set to missing and excluded from the analyses. Total unweighted denominators for exposure to tobacco product marketing included n = 9,082 (e-cigarettes) and n = 8,583 (cigarettes or other tobacco products). A composite measure of exposure to any tobacco product marketing when reading newspapers or magazines (e-cigarettes and cigarettes or other tobacco products) was assessed among respondents who had read newspapers or magazines and had nonmissing data for at least one measure (n = 10,627).

^††^ A composite measure of any advertising or promotion exposure (any source) was assessed based on exposure to retail stores; the Internet, television, streaming services, movies; and newspapers or magazines. Total unweighted denominators included n = 18,696 (e-cigarettes), n = 18,220 (cigarettes or other tobacco products), and n = 18,763 (e-cigarettes and cigarettes or other tobacco products). For each category (e-cigarettes, cigarettes or other tobacco products, and overall), persons with missing data across each potential source or those who reported “I never go to a convenience stores, supermarket, or gas station,” “I do not use the Internet,” “I do not watch TV or streaming services or go to the movies,” and “I do not read newspapers or magazines” were excluded from the analysis.

^§§^ Estimated weighted total number of students was rounded down to the nearest 10,000 persons.

^¶¶^ Hispanic persons could be of any race.

*** Respondents were instructed as follows: “The next five questions ask about issues related to e-cigarette advertisement. Do not think about cigarettes or other tobacco products when answering these questions.”

^†††^ Respondents were instructed as follows: “The next five questions ask about issues related to advertisements for tobacco products such as cigarettes, cigars, smokeless tobacco, hookahs, roll-your-own cigarettes, pipe tobacco, snus, dissolvable tobacco products, bidis, heated tobacco products, and nicotine pouches. Do not think of e-cigarettes when answering these questions.”

### E-Cigarette Content on Social Media

In total, 91.5% (23.06 million) of U.S. middle and high school students reported using social media. Among all social media users, 73.5% (high school: 80.0%; middle school: 64.5%) reported ever seeing e-cigarette-related posts or content; 13.7% (high school: 16.3%; middle school: 10.1%) reported seeing e-cigarette–related content daily (Table 10). An estimated 14.4% of social media users in middle and high school reported posting pictures or videos of e-cigarette use by themselves or others; 26.1% reported otherwise engaging (liking, commenting, or sharing) with e-cigarette–related content on social media.

**TABLE 10:** Observance of, and engagement with, e-cigarette content on social media among middle and high school students who reported using social media,* overall and by school level, sex, and race and ethnicity — National Youth Tobacco Survey, United States, 2021

Characteristic	Sex		Race and ethnicity				Total	
	Female	Male	White, non-Hispanic	Black, non-Hispanic	Hispanic^†^	Other, non-Hispanic		
	% (95% CI)	% (95% CI)	% (95% CI)	% (95% CI)	% (95% CI)	% (95% CI)	% (95% CI)	Estimated no.^§^
**Overall**								
**Seeing e-cigarette–related post or content^¶^**								
Never	23.9 (22.2–25.8)	29.1 (27.5–30.7)	23.3 (21.8–24.8)	32.3 (30.0–34.6)	28.3 (26.4–30.2)	31.1 (25.9–36.7)	**26.5 (25.2–27.9)**	**6,060,000**
Less than monthly	25.6 (24.3–26.8)	27.2 (25.7–28.7)	26.6 (25.2–28.0)	24.7 (22.6–26.8)	25.6 (24.0–27.3)	31.8 (29.0–34.6)	**26.4 (25.3–27.5)**	**6,030,000**
Monthly	14.1 (13.1–15.3)	13.2 (12.3–14.1)	14.7 (13.7–15.7)	10.5 (9.1–12.0)	13.7 (12.8–14.7)	12.1 (9.6–15.1)	**13.7 (13.0–14.3)**	**3,120,000**
Weekly	20.4 (18.8–22.2)	19.1 (17.7–20.5)	21.8 (20.1–23.5)	17.4 (15.5–19.4)	17.8 (16.2–19.5)	16.3 (13.5–19.7)	**19.7 (18.5–21.0)**	**4,510,000**
Daily	15.9 (14.5–17.4)	11.5 (10.7–12.5)	13.6 (12.3–15.1)	15.2 (13.4–17.2)	14.6 (13.4–15.9)	8.8 (6.7–11.4)	**13.7 (12.8–14.7)**	**3,130,000**
Posting pictures or videos of e-cigarette use (by self or others)**								
Never	85.8 (84.5–87.0)	85.5 (84.2–86.8)	87.4 (86.0–88.6)	80.0 (77.1–82.5)	84.0 (82.6–85.3)	87.4 (84.3–89.9)	**85.6 (84.6–86.6)**	**19,490,000**
Less than monthly	6.8 (6.0–7.6)	6.3 (5.6–7.1)	6.1 (5.4–6.8)	8.0 (6.7–9.5)	7.4 (6.3–8.7)	5.8 (3.9–8.8)	**6.6 (6.1–7.1)**	**1,490,000**
Monthly	2.4 (1.9–3.0)	3.3 (2.8–3.9)	2.5 (2.1–3.0)	4.0 (3.3–5.0)	3.1 (2.6–3.7)	3.0 (2.1–4.2)	**2.9 (2.5–3.2)**	**640,000**
Weekly	3.3 (2.8–3.9)	2.6 (2.2–3.2)	2.7 (2.1–3.4)	3.6 (2.8–4.7)	3.1 (2.6–3.7)	2.7 (1.6–4.6)	**2.9 (2.6–3.3)**	**670,000**
Daily	1.8 (1.5–2.1)	2.2 (1.8–2.8)	1.3 (1.0–1.7)	4.4 (3.3–5.9)	2.4 (2.0–3.0)	—^††^	**2.0 (1.7–2.3)**	**450,000**
**Liking, commenting, sharing e-cigarette–related posts or content^§§^**								
Never	70.4 (69.0–71.8)	77.5 (76.2–78.7)	73.6 (72.2–74.9)	72.1 (69.8–74.3)	73.6 (72.1–75.0)	79.9 (76.1–83.3)	**73.9 (72.9–74.9)**	**16,750,000**
Less than monthly	16.9 (15.8–18.1)	13.0 (11.8–14.3)	15.8 (14.8–17.0)	15.2 (13.2–17.4)	14.1 (12.7–15.6)	12.6 (10.2–15.6)	**15.0 (14.2–15.8)**	**3,390,000**
Monthly	5.2 (4.6–5.8)	4.0 (3.4–4.6)	4.7 (4.1–5.3)	3.8 (2.9–4.9)	5.2 (4.4–6.1)	3.2 (2.1–4.8)	**4.6 (4.1–5.0)**	**1,030,000**
Weekly	5.0 (4.4–5.8)	3.5 (3.1–4.0)	4.1 (3.7–4.6)	4.6 (3.9–5.6)	4.6 (4.1–5.2)	2.9 (1.9–4.3)	**4.3 (4.0–4.6)**	**960,000**
Daily	2.5 (2.0–3.1)	2.0 (1.6–2.4)	1.7 (1.4–2.2)	4.3 (3.3–5.5)	2.6 (1.9–3.4)	1.3 (0.8–2.1)	**2.3 (1.9–2.7)**	**510,000**
**High school (grades 9–12)**								
**Seeing e-cigarette–related post or content**								
Never	17.7 (15.8–19.7)	22.3 (20.8–23.9)	17.2 (15.7–18.8)	29.1 (26.1–32.3)	21.5 (19.9–23.1)	23.0 (15.8–32.1)	**20.0 (18.7–21.4)**	**2,650,000**
Less than monthly	24.3 (22.8–25.8)	27.7 (25.8–29.7)	25.9 (24.1–27.7)	22.6 (20.2–25.2)	26.9 (24.9–29.0)	32.2 (28.4–36.3)	**26.1 (24.7–27.5)**	**3,450,000**
Monthly	15.3 (13.7–17.0)	15.0 (13.6–16.5)	16.2 (14.8–17.7)	12.3 (10.4–14.6)	14.3 (12.8–15.9)	14.5 (10.7–19.4)	**15.1 (14.2–16.2)**	**2,000,000**
Weekly	23.6 (21.6–25.7)	21.3 (19.6–23.1)	24.6 (22.5–26.8)	18.3 (15.6–21.3)	19.8 (17.4–22.3)	20.7 (15.9–26.5)	**22.4 (20.9–24.0)**	**2,970,000**
Daily	19.1 (17.2–21.3)	13.7 (12.4–15.1)	16.1 (14.2–18.3)	17.7 (15.4–20.2)	17.6 (15.6–19.8)	9.5 (6.7–13.4)	**16.3 (15.0–17.7)**	**2,160,000**
**Posting pictures or videos of e-cigarette use (by self or others)**								
Never	83.9 (82.6–85.2)	84.2 (82.4–85.8)	85.9 (84.0–87.6)	79.3 (76.5–81.8)	82.0 (80.2–83.6)	85.1 (80.9–88.5)	**84.0 (82.8–85.2)**	**11,080,000**
Less than monthly	7.1 (6.2–8.1)	6.8 (5.9–7.8)	6.6 (5.6–7.7)	7.4 (5.9–9.3)	7.8 (5.7–10.6)	6.6 (4.3–10.0)	**6.9 (6.3–7.7)**	**910,000**
Monthly	2.9 (2.3–3.6)	3.8 (3.0–4.8)	2.9 (2.4–3.7)	4.5 (3.4–5.9)	3.8 (2.9–4.8)	3.3 (1.9–5.7)	**3.4 (2.9–4.0)**	**440,000**
Weekly	3.9 (3.3–4.7)	3.0 (2.5–3.6)	3.1 (2.5–3.7)	4.1 (3.0–5.5)	3.8 (3.1–4.6)	—	**3.4 (3.0–3.9)**	**450,000**
Daily	2.1 (1.7–2.7)	2.3 (1.7–3.1)	1.5 (1.1–2.1)	4.7 (3.2–6.7)	2.7 (2.1–3.5)	—	**2.2 (1.8–2.6)**	**290,000**
**Liking, commenting, sharing e-cigarette–related posts or content**								
Never	66.7 (65.0–68.4)	73.7 (72.0–75.3)	69.9 (68.0–71.8)	70.9 (67.8–73.9)	69.8 (68.0–71.4)	74.6 (68.8–79.8)	**70.3 (69.1–71.4)**	**9,230,000**
Less than monthly	19.6 (18.0–21.3)	15.6 (14.1–17.3)	18.2 (16.7–19.8)	16.7 (13.9–19.8)	17.2 (15.3–19.3)	16.3 (12.7–20.6)	**17.6 (16.5–18.7)**	**2,310,000**
Monthly	5.7 (5.0–6.6)	4.9 (4.0–5.9)	5.6 (4.9–6.6)	3.5 (2.3–5.2)	5.7 (4.9–6.7)	4.0 (2.2–7.1)	**5.3 (4.7–5.9)**	**690,000**
Weekly	5.7 (4.8–6.7)	3.9 (3.3–4.6)	4.7 (4.0–5.5)	5.3 (4.2–6.7)	4.9 (4.0–6.0)	3.7 (2.4–5.7)	**4.8 (4.2–5.4)**	**620,000**
Daily	2.2 (1.7–2.9)	1.9 (1.4–2.6)	1.6 (1.2–2.0)	3.6 (2.6–5.0)	2.4 (1.7–3.5)	—	**2.1 (1.7–2.5)**	**270,000**
**Middle school (grades 6–8)**								
**Seeing e-cigarette–related post or content**								
Never	32.2 (30.2–34.3)	39.1 (36.5–41.7)	32.8 (30.6–35.2)	37.1 (33.7–40.7)	36.9 (33.9–39.9)	38.8 (33.4–44.5)	**35.5 (33.7–37.3)**	**3,390,000**
Less than monthly	27.2 (25.4–29.0)	26.3 (24.1–28.5)	27.7 (25.7–29.8)	27.4 (24.2–30.9)	24.0 (21.6–26.5)	31.3 (27.3–35.7)	**26.7 (25.3–28.2)**	**2,550,000**
Monthly	12.6 (11.1–14.3)	10.5 (9.3–11.8)	12.3 (10.8–13.9)	7.8 (5.9–10.3)	13.0 (10.9–15.5)	9.7 (7.2–12.9)	**11.6 (10.6–12.7)**	**1,100,000**
Weekly	16.4 (14.8–18.1)	15.8 (14.0–17.7)	17.5 (15.7–19.4)	16.1 (13.9–18.6)	15.2 (13.1–17.7)	12.1 (9.5–15.2)	**16.1 (14.8–17.5)**	**1,530,000**
Daily	11.6 (10.1–13.3)	8.4 (7.1–9.8)	9.7 (8.2–11.5)	11.6 (9.2–14.4)	10.9 (9.3–12.8)	8.1 (5.5–11.8)	**10.1 (9.0–11.3)**	**960,000**
**Posting pictures or videos of e-cigarette use (by self or others)**								
Never	88.4 (86.3–90.3)	87.6 (85.8–89.2)	89.7 (88.0–91.2)	81.3 (75.8–85.7)	86.7 (84.3–88.8)	89.6 (86.2–92.2)	**88.0 (86.3–89.4)**	**8,370,000**
Less than monthly	6.2 (5.1–7.6)	5.6 (4.8–6.6)	5.3 (4.3–6.5)	8.4 (6.5–10.8)	6.9 (5.5–8.5)	—	**6.0 (5.2–6.9)**	**570,000**
Monthly	1.7 (1.2–2.4)	2.6 (2.1–3.2)	1.8 (1.4–2.4)	3.4 (2.2–5.2)	2.2 (1.5–3.3)	2.7 (1.9–3.8)	**2.1 (1.8–2.6)**	**200,000**
Weekly	2.4 (1.6–3.4)	2.1 (1.5–3.1)	2.1 (1.3–3.4)	2.9 (1.8–4.7)	2.2 (1.4–3.7)	—	**2.2 (1.6–3.0)**	**210,000**
Daily	1.3 (0.9–1.9)	2.0 (1.4–2.9)	1.0 (0.6–1.7)	4.0 (2.3–6.9)	1.9 (1.3–2.9)	—	**1.7 (1.2–2.2)**	**150,000**
**Liking, commenting, sharing e-cigarette–related posts or content**								
Never	75.2 (72.9–77.4)	83.0 (81.1–84.7)	79.3 (77.4–81.1)	73.7 (70.1–77.0)	78.4 (75.6–80.9)	85.0 (81.0–88.2)	**79.0 (77.3–80.5)**	**7,480,000**
Less than monthly	13.3 (11.9–15.0)	9.3 (8.0–10.7)	12.2 (11.0–13.6)	13.0 (11.2–15.0)	10.1 (8.3–12.4)	9.2 (6.8–12.4)	**11.4 (10.4–12.4)**	**1,070,000**
Monthly	4.4 (3.6–5.3)	2.7 (2.2–3.4)	3.2 (2.6–3.9)	4.3 (3.2–5.7)	4.5 (3.4–6.0)	2.5 (1.5–4.1)	**3.6 (3.0–4.2)**	**330,000**
Weekly	4.2 (3.4–5.1)	3.0 (2.3–3.8)	3.3 (2.7–4.1)	3.7 (2.6–5.2)	4.2 (3.4–5.3)	—	**3.6 (3.1–4.1)**	**330,000**
Daily	2.9 (2.1–3.8)	2.0 (1.4–2.9)	2.0 (1.4–2.8)	5.3 (3.3–8.5)	2.7 (1.9–4.0)	—	**2.5 (2.0–3.2)**	**230,000**

**Abbreviation: **e-cigarettes = electronic cigarettes.

*All respondents were asked, “How often do you use social media?” Response options included “never/I don’t use social media”; “less than one time per week”; “about one time per week”; “a few times per week”; “less than 1 hour, daily”; “about 1–2 hours, daily”; “about 3–4 hours, daily”; “4 hours or more, daily.” Subsequent questions on observance and engagement with e-cigarette content were assessed among those with nonmissing data who reported a response other than “never/I don’t use social media” (n = 16,960).

^† ^Hispanic persons could be of any race.

^§^ Estimated weighted total number of students was rounded down to the nearest 10,000 persons. Overall estimates might not directly total to sums of corresponding subgroup estimates because of rounding or inclusion of students who did not self-report grade level.

^¶ ^Assessed by the question, “When you use social media, how often do you see posts or content (pictures, videos, or text) related to e-cigarettes?”

^** ^Assessed by the question, “When you use social media, how often do you post pictures or videos of yourself or someone else using e-cigarettes?”

^†† ^Data were statistically unreliable because of unweighted denominator <50 or a relative standard error >30%.

^§§ ^Assessed by the question, “When you use social media, how often have you liked, comments, or shared posts or content (pictures, videos, or text) related to e-cigarettes?”

### Harm Perceptions

**FIGURE 4:**
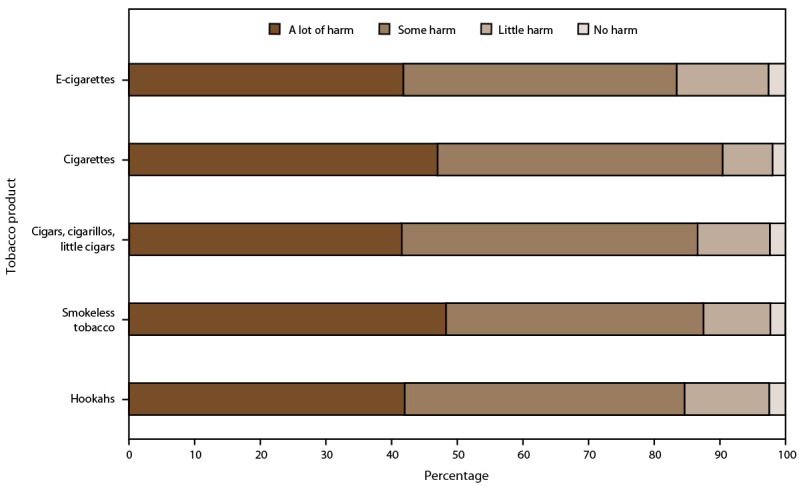
Harm perceptions of intermittent use* of select tobacco products among middle and high school students — National Youth Tobacco Survey, United States, 2021 **Abbreviation:** e-cigarettes = electronic cigarettes. * Assessed by the questions, “How much do you think people harm themselves when they [use e-cigarettes; smoke cigarettes; smoke cigars, cigarillos, or little cigars; use chewing tobacco, snuff, dip, snus, or dissolvable tobacco products; or smoke tobacco in a hookah or water pipe] some days but not every day?” Response options included “no harm,” “little harm,” “some harm,” and “a lot of harm” for each of the five tobacco products assessed. Harm perceptions of intermittent use of other tobacco products were not assessed in the 2021 National Youth Tobacco Survey.

Among all students, the percentage who reported that intermittent use of tobacco products causes a lot of harm was highest for smokeless tobacco (48.3%), followed by cigarettes (47.0%), hookahs (42.0%), e-cigarettes (41.8%), and cigars (41.5%) (Figure 4). The percentage of students who reported that intermittent use causes no harm or little harm was highest for e-cigarettes (16.6%) and lowest for cigarettes (9.6%). Estimates of harm perceptions for each tobacco product by school level are provided (Supplementary Table 3; https://stacks.cdc.gov/view/cdc/114659).

### Dependence and Cessation Indicators

Among students who currently used any tobacco product, 27.2% (high school: 27.1%; middle school: 28.1%) reported experiencing cravings for tobacco product of any kind during the past 30 days (Table 11). Overall, 19.5% (high school: 21.8%; middle school: 9.4%) of students who currently used any tobacco product reported wanting to use a tobacco product within 30 minutes of waking.

**TABLE 11:** Urges to use tobacco products and quitting behaviors among middle and high school students who reported current (past 30-day) tobacco product use,* overall and by school level, sex, and race and ethnicity — National Youth Tobacco Survey, United States, 2021

Characteristic	Past–30-day craving^†^		Within 30 minutes of waking^§^		Thinking about quitting^¶^		Past-year quit attempt**	
	% (95% CI)	Estimated no. of users^††^	% (95% CI)	Estimated no. of users	% (95% CI)	Estimated no. of users	% (95% CI)	Estimated no. of users
**Overall**	**27.2 (24.1–30.5)**	**620,000**	**19.5 (16.9–22.4)**	**440,000**	**65.3 (61.7–68.9)**	**1,320,000**	**60.2 (57.0–63.3)**	**1,200,000**
**Sex**								
Male	22.1 (18.3–26.5)	240,000	19.8 (16.2–24.0)	220,000	61.8 (56.5–66.8)	630,000	57.6 (53.4–61.7)	590,000
Female	32.1 (27.6–37.0)	370,000	19.4 (15.6–23.8)	220,000	69.4 (64.8–73.6)	680,000	63.0 (58.7–67.1)	600,000
**Race and ethnicity**								
White, non-Hispanic	30.7 (26.7–34.9)	440,000	21.5 (18.6–24.7)	310,000	66.6 (62.0–70.9)	870,000	59.8 (55.9–63.5)	770,000
Black, non-Hispanic	19.4 (13.5–27.1)	40,000	11.3 (6.4–19.2)	20,000	62.0 (52.3–70.8)	130,000	63.0 (53.4–71.6)	140,000
Hispanic^§§^	22.0 (17.1–27.9)	90,000	17.1 (11.7–24.4)	70,000	65.6 (58.1–72.4)	240,000	61.3 (52.0–69.8)	210,000
Other, non-Hispanic	21.1 (13.0–32.3)	10,000	—^¶¶^	—	54.8 (34.4–73.8)	40,000	56.7 (35.9–75.4)	40,000
**High school (Grades 9–12)**	**27.1 (23.5–31.0)**	**500,000**	**21.8 (18.8–25.1)**	**400,000**	**64.9 (60.3–69.1)**	**1,070,000**	**58.2 (54.5–61.8)**	**950,000**
**Sex**								
Male	23.1 (18.6–28.2)	210,000	21.7 (17.7–26.4)	200,000	61.8 (55.2–68.0)	530,000	55.9 (51.4–60.3)	480,000
Female	31.4 (26.4–36.9)	280,000	22.1 (17.6–27.3)	200,000	68.7 (63.7–73.3)	540,000	60.7 (55.4–65.7)	460,000
**Race and ethnicity**								
White, non-Hispanic	30.6 (26.2–35.5)	380,000	22.9 (19.7–26.3)	280,000	66.0 (60.8–70.8)	750,000	59.2 (55.0–63.2)	670,000
Black, non-Hispanic	20.0 (13.0–29.5)	30,000	14.1 (8.0–23.6)	20,000	59.6 (48.5–69.8)	100,000	57.4 (46.4–67.8)	90,000
Hispanic	17.7 (11.8–25.7)	50,000	20.4 (12.9–30.7)	50,000	65.4 (55.0–74.5)	160,000	57.6 (45.1–69.1)	130,000
Other, non-Hispanic	—	—	—	—	—	—	—	—
**Middle school (grades 6–8)**	**28.1 (22.6–34.3)**	**120,000**	**9.4 (6.5–13.4)**	**30,000**	**67.6 (60.2–74.3)**	**240,000**	**68.4 (63.1–73.2)**	**240,000**
**Sex**								
Male	17.7 (12.2–25.0)	30,000	10.3 (6.2–16.7)	10,000	62.5 (52.6–71.4)	100,000	65.8 (57.3–73.4)	100,000
Female	35.6 (26.9–45.3)	80,000	8.8 (5.1–14.5)	20,000	71.6 (61.7–79.8)	130,000	71.6 (63.5–78.5)	130,000
**Race and ethnicity**								
White, non-Hispanic	31.4 (23.2–40.9)	50,000	12.9 (8.3–19.7)	20,000	70.2 (62.2–77.1)	110,000	63.3 (55.3–70.6)	100,000
Black, non-Hispanic	—	—	—	—	71.1 (50.4–85.6)	30,000	81.9 (69.8–89.8)	40,000
Hispanic	32.1 (22.7–43.3)	40,000	—	—	67.1 (52.6–78.9)	70,000	67.7 (53.5–79.3)	70,000
Other, non-Hispanic	—	—	—	—	—	—	—	—

**Abbreviation: **e-cigarettes = electronic cigarettes.

* Any current tobacco product use was defined as use of any tobacco product (e-cigarettes, cigarettes, cigars [cigars, cigarillos, or little cigars], smokeless tobacco [chewing tobacco, snuff or dip; snus; or dissolvable tobacco products], hookahs, pipe tobacco, heated tobacco products, nicotine pouches, or bidis [small brown cigarettes wrapped in a leaf]) on ≥1 day during the past 30 days.

^†^ Assessed by the question, “During the past 30 days, have you had a strong craving or felt like you really needed to use a tobacco product of any kind?” The response options were “yes” or “no.”

^§^ Assessed by the question, “How soon after you wake up do you want to use a tobacco product?” Response options were dichotomized as within 30 minutes (“within 5 minutes” or “from 6 to 30 minutes”) or not within 30 minutes (“from more than 30 minutes to 1 hour”; “after >1 hour but <24 hours”; “I rarely want to use tobacco products”; or “I do not want to use tobacco products”).

^¶^ Assessed by the question, “Are you seriously thinking about quitting the use of all tobacco products?” Response options were dichotomized as yes (“yes, during the next 30 days”; “yes, during the next 6 months”; “yes, during the next 12 months”; or “yes, but not during the next 12 months”) or no (“no, I am not thinking about quitting the use of all tobacco products”).

** Assessed by the question, “During the past 12 months, how many times have you stopped using all tobacco products for one day or longer because you were trying to quit all tobacco products for good?” Response options were dichotomized as yes (“1 time,” “2 times,” “3 to 5 times, “6 to 9 times,” or “10 or more times”) or no (“no, I did not try to quit all tobacco products during the past 12 months”).

^††^ Estimated weighted total numbers of users were rounded down to the nearest 10,000 persons. Overall estimates might not directly total to sums of corresponding subgroup estimates because of rounding or inclusion of students who did not self-report sex, race and ethnicity, or grade level.

^§§^ Hispanic persons could be of any race.

^¶¶^ Data were statistically unreliable because of unweighted denominator <50 or a relative standard error >30%.

Among students who currently used any tobacco product, 65.3% (high school: 64.9%; middle school: 67.6%) reported that they were seriously thinking about quitting all tobacco products. In addition, 60.2% (high school: 58.2%; middle school: 68.4%) of current tobacco product users overall reported that they stopped using all tobacco products for ≥1 day during the past 12 months because they were trying to quit.

## Discussion

### Public Health Implications

Findings from the 2021 NYTS indicate that approximately one in four students (24.1%), including approximately one in three high school students (34.0%) and one in nine middle school students (11.3%), had ever used a tobacco product. Furthermore, approximately one in 10 students (9.3%), including more than one in eight high school students (13.4%) and one in 25 middle school students (4.0%), had used a tobacco product during the past 30 days. Approximately three in 10 students (29.0%) who currently used any tobacco product reported using two or more tobacco products during the past 30 days; youths who use multiple tobacco products are at higher risk for developing nicotine dependence and might be more likely to continue using tobacco into adulthood (*1*,*2*). Most youths who currently use tobacco products are not daily users (*8*–*10*). However, even infrequent tobacco product use can lead to symptoms of nicotine dependence (*11*). Additionally, in 2021, approximately four in 10 youths who currently used e-cigarettes, and more than 20% of youths who currently used cigars, chewing tobacco, snuff, or dip, snus, and HTPs, used them frequently (on ≥20 of the past 30 days). Furthermore, for the first time in 2021, NYTS documented use of nicotine pouches among youths, an emerging tobacco product which contains nicotine powder that dissolves in the mouth without spitting. Nicotine pouches do not contain any tobacco leaf, are available in various flavors, deliver high levels of nicotine by way of nicotine salts, and have had rapidly increasing sales in recent years (*12*). Although youths’ use of nicotine pouches was low in 2021, these baseline data are needed for future monitoring to inform local, state, and federal public health policy and practice. Youth use of tobacco products in any form is unsafe, regardless of whether the products are combustible, smokeless, or electronic (*2*,*3*). Continued efforts are warranted to prevent and reduce all forms of tobacco product use among U.S. youths.

This study highlighted disparities in tobacco product use among U.S. youths. Current use of any tobacco product, overall, was highest among students who were non-Hispanic White, followed by non-Hispanic Black, Hispanic, and students of non-Hispanic other race. However, among all race and ethnicity groups, non-Hispanic Black students reported the highest prevalence of current combustible tobacco product use (5.2%), and specifically cigar use (3.1%). While youth use of tobacco products in any form is unsafe (*2*), the toll of death and disease from tobacco product use in the United States is primarily caused by cigarettes and other combustible tobacco products (*1*). In addition, use of any tobacco product was highest among students who identified their sexual identity as LGB than those who identified as heterosexual; among those identifying as transgender than those not transgender; and among those reporting increased symptom severity of psychological distress. Furthermore, although peer use and curiosity were the most commonly cited reasons for initiating e-cigarette use among ever users in 2021, among youths who currently used e-cigarettes, the most commonly cited reasons for use were attributable to feelings of anxiety, stress, or depression and the “high or buzz” associated with nicotine use. This is particularly concerning given the bidirectional association between nicotine use and mental health disorders such as depression and anxiety; studies found that youths with mental health disorders were at increased risk for cigarette smoking, but also that youth nicotine exposure was associated with the development of mental health disorders (*3*,*13*). Additionally, nicotine withdrawal is commonly accompanied by symptoms of anxiety and depression, and relief of these symptoms through use of a nicotine-containing tobacco product or nicotine replacement therapy might perpetuate continued nicotine use (*1*). Thus, the continued implementation of population-based strategies, coupled with tobacco product regulation by FDA, are critical to prevent and reduce the use of all forms of tobacco products among all youths (*1*–*3*,*14*).

Since 2014, e-cigarettes have been the most commonly used tobacco product among U.S. youths (*8**,**15**–**17*). In 2021, youth e-cigarette use remains a serious public health concern, as approximately one in nine high school students (11.3%, 1.72 million) and one in 35 middle school students (2.8%, 320,000) had used e-cigarettes during the past 30 days. While this appears lower than previously reported estimates of e-cigarette use in 2020 (*17*), any comparison of results between survey years, including the direct attribution of any potential changes in tobacco product use, is not possible because of the modifications to the fielding procedures in 2021 as a result of the COVID-19 pandemic. Specifically, approximately half of students taking the 2021 NYTS reported doing so from outside of a school building or classroom; students participating in a school building or classroom reported a higher prevalence of ever and current use of any tobacco product, including e-cigarettes (*18*), compared with students participating at home or some other place. However, declines in e-cigarette use during the COVID-19 pandemic compared with a prepandemic period have been observed in other studies of young persons (*19*,*20*). Differences in tobacco product use by survey completion setting might be caused by potential underreporting of behaviors, reduced access to tobacco products while at home (*19*,*20*), or other unmeasured characteristics among students participating outside of the classroom. Thus, the 2021 NYTS results cannot be compared with previous NYTS survey results that were primarily conducted on school campuses.

The 2021 NYTS offers insights into factors known to influence tobacco product use among youths. Overall, approximately eight in 10 students who used a tobacco product reported using a flavored product, which can increase the appeal and ease of use for youths (*2*,*3*). In addition, tobacco product marketing through traditional sources (i.e., media, Internet, print, and point of sale) and social media might prompt tobacco product initiation and use among youths (*2*,*3*,*21*,*22*); approximately 76% of students in 2021 reported exposure to tobacco product marketing through traditional sources and approximately 74% of students who used social media had seen e-cigarette–related posts or content. However, these data indicate that approximately three in four U.S. students had seen or heard at least one public education campaign against tobacco use during the preceding year, which can be an effective, evidence-based tool to educate youths about the risks of tobacco product use and to prevent initiation (*2*,*23*,*24*). Thus, tailored interventions and services could support cessation behaviors among youths who currently use tobacco products.

### Public Health Action

Sustained implementation of population-based strategies, coupled with regulation of tobacco products by FDA, is critical to preventing and reducing all forms of tobacco product use among youths (*1*–*3*,*14*). Strategies to reduce tobacco product use and initiation among all youths include increasing prices of tobacco products; establishing comprehensive clean indoor air policies that denormalize tobacco use and protect persons from exposure to secondhand smoke and e-cigarette aerosol; sustaining media campaigns that warn about the dangers of tobacco product use; reducing youth access to tobacco products, including enforcement of the federal Tobacco 21 policy^¶^; and restricting the sales of flavored e-cigarettes (*1*,*14*,*16*,*25*).

Everyone can help protect youths from the harms of tobacco products, including e-cigarettes (*3*,*14*). Parents, educators, and youth advocates can learn about the different types of tobacco products available, including various e-cigarette devices that appeal to youths. They can also set a positive example by being tobacco-free themselves and communicating the harms of tobacco product use for youths, including nicotine’s effects on adolescent brain development and addiction (*3*). Schools can adopt and enforce tobacco-free campus policies that prohibit use of all tobacco products, including but not limited to e-cigarettes. Also, schools can implement policies to not use tobacco industry–sponsored prevention programs; no tobacco industry–sponsored prevention programs that have been studied have been shown to be effective (*2*). In addition, health care providers can ask about the use of all tobacco products, including e-cigarettes, when screening patients for tobacco product use and assist those who want to quit using tobacco products (*26*).

## Limitations

The findings in this report are subject to at least three limitations. First, because of the implementation of emergency COVID-19 protocols across the country during the 2021 NYTS fielding, the survey was administered online to allow participation by eligible students at home, school, or somewhere else. Previous research has shown that the prevalence of tobacco use might differ by interview setting (*27*) and thus the 2021 NYTS results cannot be compared with previous NYTS survey results that were primarily conducted on school campuses. Second, data were self-reported and might be subject to recall and response bias. However, the validity of self-reported tobacco product use is consistently high in population-based studies overall (*28*). Finally, data were collected only from middle and high school students who attended public or private schools; findings might not be generalizable to youths who are home schooled, have dropped out of school, are in detention centers, or are enrolled in alternative schools. However, data from the Current Population Survey indicate that approximately 96% of U.S. youths aged 10–17 years were enrolled in a traditional school in 2020 (*29*).

## Conclusion

NYTS is the only comprehensive, nationally representative cross-sectional survey of U.S. middle and high school students focused exclusively on tobacco product use behaviors and associated factors. The 2021 NYTS was fully conducted amid the global COVID-19 pandemic, during which time eligible students could participate in classrooms, at home, or some other place. Findings from 2021 NYTS indicate that youth tobacco product use remains a public health threat, with approximately one in four students (24.1%) ever using a tobacco product, and approximately one in 10 students (9.3%) using a tobacco product during the past 30 days. Furthermore, disparities in tobacco product use continue to exist among population subgroups. In 2021, any tobacco product use was higher among students who identified as LGB than heterosexual; among students who identified as transgender than those not transgender; and among students who reported increased symptom severity of psychological distress. Multiple factors continue to promote tobacco product use and initiation among youths, including flavors (such as menthol), marketing, and misperceptions of harm. The comprehensive and sustained implementation of population-level evidence-based tobacco control strategies, combined with FDA’s regulation of tobacco products, is important for preventing and reducing all forms of tobacco product use among all U.S. youths. In addition, as the tobacco product marketplace continues to diversify, surveillance among youths for all forms of tobacco product use and associated factors is important to the development of public health policy and action at the national, state, and community levels.

## Footnotes

* The term “tobacco” as used in this report refers to commercial tobacco products and not to sacred and traditional use of tobacco by some American Indian communities.

† In 2019, NYTS transitioned from a paper and pencil questionnaire to an electronic survey. The 2019 and 2020 NYTS were conducted offline using provided tablet computers in schools; data collection was supported by trained survey administrators (*4*). This methodology was not feasible for the 2021 NYTS because of the implementation of emergency COVID-19 protocols across the country, including various school instructional models (in-person, distance/virtual, and hybrid), restricted visitor access at schools, travel restrictions, and other factors.

§ 45 C.F.R. part 46; 21 C.F.R. part 56.
